# Methyl eugenol attenuates age-associated oxidative fragility by coupling Ca^2+^-calpain inhibition with Band 3 stabilization in human erythrocytes

**DOI:** 10.3389/fphys.2026.1796160

**Published:** 2026-04-20

**Authors:** Jiasi Zhang, Qingwen Li, Yilan Dai, Shuaiheng Hou, Xuan Peng, Ji Zhang, Nianqiao Gong

**Affiliations:** 1 Department of Breast Surgery, The Tenth Affiliated Hospital, Southern Medical University (Dongguan People's Hospital), Dongguan, Guangdong, China; 2 Institute of Organ Transplantation, Tongji Hospital, Tongji Medical College, Huazhong University of Science and Technology; Key Laboratory of Organ Transplantation, Ministry of Education; NHC Key Laboratory of Organ Transplantation; Key Laboratory of Organ Transplantation, Chinese Academy of Medical Sciences; Organ Transplantation Clinical Medical Research Center of Hubei Province, Wuhan, Hubei, China; 3 Department of Laboratory Medicine, Dongguan Institute of Clinical Cancer Research, The Tenth Affiliated Hospital of Southern Medical University (Dongguan People's Hospital), Dongguan, Guangdong, China; 4 Department of Urology, The First Affiliated Hospital of Anhui Medical University, Institute of Urology & Anhui Province Key Laboratory of Genitourinary Diseases, Anhui Medical University, Hefei, Anhui, China

**Keywords:** Band 3 protein, calpain, erythrocyte senescence, methyl eugenol, oxidative stress

## Abstract

**Background:**

Human erythrocytes serve as an ideal model for cellular aging, a process where longevity relies on membrane scaffold integrity. The oxidative deterioration of Band 3, a major integral membrane protein, is a central driver of this senescence. This study investigated whether methyl eugenol (ME) stabilizes Band 3 against age-associated oxidative fragility.

**Methods:**

Erythrocytes were challenged with H_2_O_2_ to simulate age-associated oxidative injury. Damage was evaluated via hemolysis assays, SEM, and flow cytometry. Sulfate (SO_4_^2-^) uptake kinetics and Western blotting were employed to assess Band 3 anion exchange function and structural stability. *In silico* docking simulated interactions between ME metabolites and the Band 3 structure. Physiological relevance was validated in a human cohort (n = 81; 20–90 years) via regression and stratified analyses of glutathione (GSH) and malondialdehyde (MDA) levels.

**Results:**

ME exhibited an optimal protective concentration at 2 µM, effectively preserving biconcave morphology and attenuating hemolysis. Treatment significantly mitigated intracellular oxidative stress and rescued cell viability. Mechanistically, ME suppressed the pathological increase in intracellular Ca^2+^ concentration and inhibited calpain activity. Functionally, ME significantly restored sulfate transport rates. Western blotting confirmed that ME specifically preserved the full-length (100 kDa) and cytoplasmic (43 kDa) domains of Band 3, whereas the 55 kDa transmembrane domain remained largely unaffected. Docking simulations predicted a specific interaction with residue ARG292 within the cytoplasmic domain, suggesting a structural basis for this stabilization. In the donor cohort, ME extended the projected GSH half-life (from 47.14 to 64.14 years) and reduced maximal lipid peroxidation by ~40%.

**Conclusion:**

ME mitigates oxidative eryptosis by coupling Ca^2+^–calpain inhibition with site-specific Band 3 stabilization, offering a rationale for using ME to standardize erythrocyte quality and reduce age-associated fragility.

## 1 Introduction

Human erythrocytes represent an ideal model for studying cellular aging because of their finite lifespan, well-defined biochemical properties, and high susceptibility to cumulative oxidative injury (Yu et al., 2025). During their approximately 120-day circulatory lifespan, erythrocytes are continuously exposed to oxidative stress from intrinsic and extrinsic sources. However, they lack the capacity for protein synthesis or genomic repair (D’Alessandro, 2025); thus, their functional longevity is strictly contingent upon the structural integrity of the membrane scaffold and the resilience of endogenous antioxidant systems. Erythrocyte senescence represents a multidimensional process characterized by progressive metabolic attrition, particularly redox imbalance and membrane degradation (Brun et al., 2022). This progression is accompanied by hallmark features, including the transition to an echinocytic phenotype (Savitskaya et al., 2022), a shift toward a pro-oxidant state indicated by glutathione (GSH) depletion and accumulation of malondialdehyde (MDA) (Singh et al., 2025), and externalization of phosphatidylserine (PS) (Kao et al., 2025), which serves as an “eat-me” signal for splenic clearance. These age-related changes are further accelerated under pathological conditions and during blood storage, contributing to the development of the “storage lesion” and ultimately compromising transfusion efficacy (Peltier et al., 2025). Therefore, identifying agents that decelerate erythrocyte functional decline by enhancing structural resilience is of considerable translational relevance.

A pivotal event driving erythrocyte senescence is the oxidative destabilization of Band 3 (Vani et al., 2024), a major integral erythrocyte membrane protein. Band 3 comprises a C-terminal transmembrane domain that mediates anion exchange and an N-terminal cytoplasmic domain of approximately 43 kDa that anchors the spectrin-based cytoskeleton (Tkachenko et al., 2025). Despite its critical structural role, this cytoplasmic domain is vulnerable to age-associated proteolytic cleavage, a process that destabilizes the membrane-cytoskeleton interface and triggers vesiculation (Jin et al., 2024). Accordingly, stabilization of the Band 3 structural scaffold is widely recognized as a critical therapeutic target for retarding erythrocyte senescence (Kyriacou and Shibeeb, 2025).

This proteolytic degradation is inextricably linked to intracellular Ca^2+^ dysregulation. The terminal clearance of senescent erythrocytes often occurs through eryptosis, a Ca^2+^-dependent process (Dreischer et al., 2022). Under oxidative stress, Ca^2+^ channels are activated, leading to a sustained increase in cytosolic Ca^2+^ levels (Sikandar et al., 2025). This Ca^2+^ influx triggers PS externalization and activates calpain, which further degrades cytoskeletal proteins, including Band 3 (Qiao, 2025). This establishes a deleterious feed-forward loop: oxidative stress triggers Ca^2+^ influx, which activates calpain, leading to Band 3 cleavage and membrane fragility. Consequently, an effective protective strategy must simultaneously mitigate Ca^2+^ dysregulation and stabilize Band 3—a stringent requirement that many conventional antioxidants do not adequately fulfill.

To identify potential protective agents, we examined methyl eugenol (ME; 1,2-dimethoxy-4-prop-2-enylbenzene), a naturally occurring phenylpropanoid widely present in the essential oils of medicinal plants such as *Syzygium aromaticum* (clove) and *Asarum* species (wild ginger) (Tan and Nishida, 2012). Its long-standing use as a principal component of a clinical wound-healing ointment suggests a favorable preliminary safety profile (Nianqiao, 2014). Consistent with this, our previous study revealed that ME confers protection against ischemia–reperfusion injury *in vivo* (Kuang et al., 2023). Structurally, ME possesses potential pharmacophore features that may facilitate specific molecular interactions with protein domains (Api et al., 2023). We hypothesized that, at appropriate concentrations, ME acts not only as a radical scavenger but also facilitates site-specific molecular interactions with the N-terminal domain of Band 3.

In the present study, using human erythrocytes as a translational model, we investigated the protective efficacy of ME against oxidative fragility. By integrating mechanistic dissection of the Ca^2+^-calpain-Band 3 axis with a cross-sectional analysis of a donor cohort, we aimed to elucidate ME’s capacity to couple metabolic stability with structural preservation, thereby establishing its translational relevance for mitigating age-associated functional decline.

## 2 Materials and methods

### 2.1 Solutions and chemicals

ME (S5755, Selleck Chemicals, Houston, TX, USA) was dissolved in dimethyl sulfoxide (DMSO; BL165A, Biosharp, Hefei, China) and serially diluted to working concentrations ranging from 0.1 to 1000 µM. This wide screening range was selected based on previous *in vitro* toxicological and pharmacological evaluations to comprehensively define its therapeutic window and potential high-dose toxicity (Burkey et al., 2000; Kuang et al., 2023). Hydrogen peroxide (H_2_O_2_; H325-100, Sinopharm, Shanghai, China) was freshly prepared by dilution from a 30% (w/w) stock solution. The catalase inhibitor 3-amino-1,2,4-triazole (3-AT; A8056, Sigma-Aldrich, St. Louis, MO, USA) was dissolved in distilled water to obtain a 10 mM stock solution. The Band 3–specific inhibitor 4,4’-diisothiocyanatostilbene-2,2’-disulfonate (DIDS; D3514, Sigma-Aldrich, St. Louis, MO, USA) was dissolved in 0.1 M KHCO_3_ buffer to prepare a 10 mM stock solution. Ionomycin (HY-13434; MedChemExpress, Monmouth Junction, NJ, USA) was prepared in ethanol from a 14.1 mM stock solution. EDTA (E5134; Sigma-Aldrich, St. Louis, MO, USA) was prepared at 0.5 mM in ultrapure water (pH 8.0) and stored until use.

### 2.2 Ethical approval and study population

All blood samples were collected and processed in accordance with protocols approved by the Institutional Review Board of Tongji Hospital, Tongji Medical College, Huazhong University of Science and Technology (approval date: April 6, 2019; approval no. TJ-IRB20190406). This overarching ethical protocol covers research related to body fluids and cellular aging. Because this study exclusively utilized de-identified, leftover peripheral blood samples remaining after routine clinical laboratory testing, the requirement for written informed consent was officially waived by the IRB, as documented in the approval protocol. All procedures complied with the ethical principles of the Declaration of Helsinki.

To ensure strict adherence to the exclusion criteria while maintaining donor anonymity, clinical and demographic data (including medical history, medication use, and lifestyle factors) were derived from standard pre-examination questionnaires routinely administered by the Health Management Center. An authorized clinical data manager applied the exclusion criteria to select eligible sample barcodes, and only the corresponding de-identified leftover samples were provided to the research team. Participants were excluded if they met any of the following criteria: (1) current use of any medication or oral antioxidant supplements; (2) active smoking within the past 3 months; (3) presence of common acute or chronic illnesses, such as diabetes, tuberculosis, or asthma; (4) diagnosis of serious conditions, including infectious, cardiovascular, or hematological diseases; or (5) pregnancy or lactation in female donors. After applying these criteria, the final study cohort comprised 81 healthy donors aged 20–90 years, with samples collected during the active validity period of the protocol.

### 2.3 Erythrocyte preparation

Peripheral blood samples were obtained as de-identified, leftover specimens from routine health examinations at the Health Management Center. Because these were routine physical check-ups, the blood was originally collected into lithium heparin tubes between 8:00 and 10:00 AM following a 12-h overnight fast to preserve baseline physiological extracellular calcium levels. To ensure sample freshness and high viability, the leftover erythrocytes were retrieved immediately following the completion of the clinical laboratory assays (within 2–4 hours of the initial blood draw).

To prepare packed erythrocytes, the collected whole blood was centrifuged at 3000 × g for 10 min at 4 °C. Plasma and the buffy coat were removed along with the upper 10%–25% of the erythrocyte layer. Erythrocytes were then washed three times at 1000 × g for 5 min at 4 °C with an isotonic buffer (145 mM NaCl, 5 mM glucose, and 20 mM HEPES; pH 7.4, 300 mOsm) (Liu et al., 2025; Madias et al., 2024) to remove residual plasma and leucocytes. Washed erythrocytes were subsequently suspended to 3% hematocrit in isotonic buffer supplemented with either DMSO (vehicle control) or ME, in the presence or absence of 300 µM H_2_O_2_ as indicated for specific assays. To ensure a sufficient absolute cell yield for downstream applications, treatments were performed in working volumes of 1 mL or greater (e.g., 30 µL of packed erythrocytes suspended in 970 µL of buffer), yielding an exposure density of approximately 3 × 10^5^ cells/µL. The suspensions were incubated in the dark at 37 °C for 24 h with gentle shaking. This temperature was selected to maintain physiological enzymatic kinetics, while the 24-hour acute exposure window was optimized to reliably induce the complete eryptotic signaling cascade without triggering non-specific spontaneous ex vivo hemolysis, a protocol consistent with established eryptosis models (Shan et al., 2016; Remigante et al., 2022a). Minor interdonor and intradonor variability in baseline erythrocyte viability was observed; therefore, all comparative treatments within a given experiment were performed using erythrocytes derived from the same donor.

### 2.4 H_2_O_2_ treatment

To simulate the accelerated oxidative stress associated with erythrocyte aging *in vitro*, a well−established H_2_O_2_ exposure system was used (Lutz and Bogdanova, 2013). This model reproduces key characteristics of aged erythrocytes, including the morphological transition from discocytes to echinocytes, oxidative modification of membrane proteins such as Band 3, and induction of eryptosis (Mohanty et al., 2014). To assess the protective effects of ME against age−related oxidative injury, erythrocytes were incubated with 300 μM H_2_O_2_ at 37 °C for 24 h with gentle shaking. DMSO (vehicle control) or ME was added concurrently to the corresponding treatment groups.

### 2.5 Hemolysis assays

The hemolytic rate was determined as previously described (Liu et al., 2025). Briefly, erythrocytes were treated with or without ME at various concentrations for 24 h under gentle shaking. After treatment, 200 μL of supernatant from each sample was collected to quantify hemoglobin release by measuring absorbance at 415 nm (A_t_). Baseline hemolysis (0%, A_0_) and complete hemolysis (100%, A_p_c) controls were generated using phosphate-buffered saline (PBS) and 1% Triton X-100, respectively. The percentage of hemolysis (Z) was calculated using the following formula:


$$Z\;\frac{A_{t}}{A_{p}c}\;\times \;100\%$$

### 2.6 Light microscopy

Following treatment with ME and H_2_O_2_, 10 µL of the erythrocyte suspension (3% hematocrit) was placed onto a microscope slide and gently covered with a coverslip to create an even monolayer, as previously described (Deniz et al., 2023). The prepared slides were then examined using a light microscope (ECLIPSE Ci, Nikon, Tokyo, Japan).

### 2.7 Scanning electron microscopy

For scanning electron microscopy (SEM), erythrocytes treated with DMSO or ME were washed with PBS and fixed in 2.5% glutaraldehyde for 1 h at room temperature with gentle shaking. Cells were then centrifuged at 1000 × *g*, washed again with PBS, and resuspended. After mounting onto glass slides, samples were dehydrated through a graded ethanol series (50%, 70%, 90%, and 100%) and air-dried using a critical-point dryer (K850, Quorum Technologies, Laughton, UK). The dried samples were subsequently sputter-coated with a thin layer of gold–palladium and examined using a SEM (Regulus 8100, HITACHI, Tokyo, Japan) at magnifications of 2000× and 9000×, as previously described (Loyola-Leyva et al., 2022), with minor modifications.

### 2.8 Flow cytometry

PS exposure was quantified by Annexin V signaling using an Annexin V−PE Apoptosis Detection Kit (C1065S, Beyotime Biotechnology, Shanghai, China). Briefly, erythrocytes were washed twice with PBS and resuspended in binding buffer at a density of 1 × 10^7^ cells/mL. A 100 µL aliquot of the suspension was then incubated with 5 µL of Annexin V−PE for 15 min at room temperature in the dark. After incubation, 400 µL of binding buffer was added, and samples were immediately analyzed using flow cytometry (NovoCyte Quanteon, Agilent, Santa Clara, CA, USA). Intact erythrocytes were gated based on forward− and side−scatter properties to exclude debris and aggregates. Within this gate, cells with positive Annexin V−PE fluorescence were quantified as eryptotic and reported as the percentage of Annexin V^+^ erythrocytes.

Intracellular esterase activity and metabolic viability were evaluated using the fluorescent dye calcein−AM, analogous to the calcein−AM/PI assay for nucleated cells (Wu et al., 2025). Following treatment, erythrocytes were incubated with calcein−AM (C2012, Beyotime Biotechnology, Shanghai, China) for 20 min at room temperature. Samples were then immediately analyzed using flow cytometry, and the proportion of calcein−AM^+^ cells was recorded as the metabolically active and viable cell fraction.

### 2.9 Generation of reactive oxygen species

Intracellular reactive oxygen species (ROS) production was assessed using the 2’,7’-dichlorodihydrofluorescein diacetate (DCFH-DA) assay (Tsamesidis et al., 2020) with slight modifications, using a Reactive Oxygen Species Assay Kit (S0033S, Beyotime Biotechnology, Shanghai, China). After treatment, erythrocytes (3% hematocrit) were incubated with 10 μM DCFH-DA for 30 min at 37 °C. Cells were then centrifuged at 1000 × *g* for 5 min at 4 °C, washed to remove excess DCFH-DA, and resuspended in PBS to a final hematocrit of 10%. Finally, flow cytometry was used to determine the average fluorescence intensity of each sample, and data are reported as the mean fluorescence intensity (MFI) of DCFH-DA.

### 2.10 Lipid peroxidation

MDA levels were measured using a Lipid Peroxidation MDA Assay Kit (S0131M, Beyotime Biotechnology, Shanghai, China) according to the manufacturer’s instructions. Following washing and suspension of erythrocytes at 3% hematocrit, erythrocytes were incubated for 24 h with either 2 µM ME or DMSO at 37 °C with gentle shaking in the dark. Lipid peroxidation was quantified by measuring the absorbance at 532 nm of the pink-colored complex formed by the reaction between thiobarbituric acid and MDA using a microplate reader (Infinite E Plex, Tecan, Männedorf, Switzerland), as previously described (Saidane et al., 2026), with minor modifications.

### 2.11 Reduced GSH

Reduced GSH levels were measured using a Reduced GSH Content Assay Kit (BC1175, Solarbio, Beijing, China) following the manufacturer’s instructions. Erythrocytes were washed, resuspended at 3% hematocrit, and incubated for 24 h with 2 µM ME at 37 °C under gentle shaking in the dark. As described previously (Boşgelmez et al., 2025), GSH levels were quantified based on the formation of 2-nitro-5-thiobenzoic acid, a yellow-colored complex formed from the reaction between 5,5’-ditiobis(2-nitrobenzoic acid) and reduced GSH. Absorbance was measured at 412 nm using a microplate reader (Infinite E Plex, Tecan, Männedorf, Switzerland).

### 2.12 Erythrocyte membrane preparation

Erythrocyte membranes were prepared as previously described (Perrone et al., 2025), with minor modifications. Following treatment with 300 μM H_2_O_2_ in the presence or absence of ME, erythrocyte membrane proteins were isolated using cold hemolysis buffer (2.5 mM NaH_2_PO_4_) supplemented with protease and phosphatase inhibitors (1 mM PMSF, 1 mM NaF, and 1 mM sodium orthovanadate). Packed cells were diluted in buffer and centrifuged repeatedly (18,000 × *g*, 10 min, 4 °C) to remove hemoglobin. The resulting membrane pellet was solubilized in 1% (v/v) sodium dodecyl sulfate (SDS) and incubated on ice for 20 min. The supernatant containing solubilized proteins was collected, and protein concentration was determined using the BCA assay (P0012, Beyotime Biotechnology, Shanghai, China) according to the manufacturer’s instructions. The samples were then stored at −80 °C until further analysis.

### 2.13 Western blotting

Equal amounts of protein were separated by 10% SDS–polyacrylamide gel electrophoresis and transferred to polyvinylidene fluoride membranes. Membranes were blocked with 5% nonfat milk for 2 h at 25 °C and incubated overnight at 4 °C with primary antibodies against Band 3 (18566-1-AP, Proteintech, Wuhan, China) and β-actin (66009-1-Ig, Proteintech, Wuhan, China). After washing with Tris-buffered saline containing 0.1% Tween-20 (TBST), membranes were incubated with horseradish peroxidase–conjugated secondary antibodies (SA00001-2, Proteintech, Wuhan, China) for 1 h at 25 °C. Membranes were washed four times with TBST (5 min each) and visualized using an Omni-ECL Ultra-Sensitive Chemiluminescence Detection Kit (SQ201, EpiZyme, Shanghai, China). Band intensities were quantified by densitometric analysis using ImageJ software (National Institutes of Health, Bethesda, MD, USA), and all results were normalized to β-actin. All Western blot experiments and subsequent densitometric quantifications were performed using three independent biological replicates (n = 3).

### 2.14 SO_4_^2−^ uptake measurement

Band 3–mediated SO_4_^2−^ uptake was assessed using a turbidimetric assay (Romano and Passow, 1984). Following treatment with 0.1% (v/v) DMSO or 2 µM ME for 24 h at 37 °C, erythrocytes were suspended at 3% hematocrit in SO_4_^2−^-containing solution (118 mM Na_2_SO_4_, 10 mM HEPES, and 5 mM glucose; pH 7.4, 300 mOsm). Uptake was terminated at specified time points by the addition of 10 µM DIDS. Cells were washed, lysed in distilled water, and treated with 4% perchloric acid to hydrolyze proteins. After centrifugation (2,500 × *g*, 10 min, 4 °C), SO_4_^2−^ in the supernatant was precipitated by sequential addition of a glycerol/water solution, an NaCl/HCl mixture, and BaCl_2_. The absorbance of the resulting precipitate was measured at 425 nm using a microplate reader (Infinite E Plex, Tecan, Männedorf, Switzerland).

A calibrated standard curve, generated by precipitating known concentrations of SO_4_^2−^, was used to convert absorbance values into intracellular SO_4_^2−^ concentrations, expressed as μmol/L cells × 10^−2^. The uptake rate constant (*r*, min^−1^) was calculated using the following equation:


$$C_{t}\;=\;C_{\infty }\;\left(1\;-e^{-irt}\right)+\;C_{0}$$

where *t* is the time of sample collection; *C_t_*, *C_0_*, and *C_∞_* represent intracellular SO_4_^2−^ concentrations at times *t*, *0*, and *∞*, respectively; *e* is the base of the natural logarithm (≈ 2.718); and *r* is the rate constant accounting for the velocity of the uptake process. The rate constant corresponds to the time required to reach 63% of the total intracellular SO_4_^2−^ concentration. In figures, SO_4_^2−^ is expressed as the micromolar concentration of sulfate trapped per 1 mL of erythrocytes at 3% hematocrit (Remigante et al., 2022c).

### 2.15 Calpain activity assay

Calpain activity was measured as previously described (Madias et al., 2024). Following treatment, erythrocytes were washed twice with PBS (pH 7.4) and counted to normalize cell density. Cells were then lysed in lysis buffer to release intracellular contents. Calpain activity was measured using a commercial fluorometric assay kit (P0375, Beyotime Biotechnology, Shanghai, China) according to the manufacturer’s instructions. Briefly, erythrocyte lysates were incubated with the calpain-specific substrate Ac-LLY-AFC, and fluorescence intensity was monitored kinetically at excitation/emission wavelengths of 400/505 nm from 0 to 120 min at 15-min intervals using a microplate reader (Infinite E Plex, Tecan, Männedorf, Switzerland).

The fluorescence ratio (emission/excitation, 505/400 nm) was calculated at each time point to correct for background interference. Initial reaction velocity was determined from the linear phase of the kinetic curve using linear regression. Calpain activity was expressed as a percentage relative to untreated control, which was defined as 100%.

### 2.16 Molecular docking

The crystal structure of Band 3 was retrieved from the Protein Data Bank (PDB; https://www.rcsb.org/; PDB ID: 1HYN). Two-dimensional (2D) structures of the chemical compounds were obtained from PubChem (https://pubchem.ncbi.nlm.nih.gov/). Molecular docking simulations were conducted using Schrödinger 2023 software (Schrödinger, New York, NY, USA) (Han et al., 2023). Following generation of ligand–protein complex conformations, binding interactions were validated through pose analysis, and detailed 2D interaction diagrams were generated. All molecular visualization and rendering were performed using PyMOL v2.3.0 (open-source PyMOL, Schrödinger, New York, NY, USA) in accordance with standardized graphical presentation guidelines.

### 2.17 Statistical analysis

All statistical analyses were conducted using GraphPad Prism 10 (GraphPad Software, Boston, MA, USA), and results are reported as the mean ± standard deviation. Unless otherwise specified, *n* represents the number of independent biological replicates (i.e., experiments performed using distinct blood samples from different donors). Specifically, all Western blot experiments and subsequent densitometric quantifications were performed using three independent biological replicates (*n* = 3). For each biological replicate, measurements were performed in triplicate to serve as technical replicates. Before hypothesis testing, data normality and variance homogeneity were validated using the Shapiro–Wilk and Brown–Forsythe tests, respectively. Intergroup differences were evaluated using Student’s *t*-test or one-way/two-way ANOVA, followed by Tukey’s *post-hoc* test. To characterize age-dependent kinetics across the donor cohort (N = 81; young, n = 40; middle-aged, n = 16; and elderly, n = 25), GSH concentrations were modeled using one-phase exponential decay, whereas MDA levels were fitted with Gompertz growth regression. Differences in fitting parameters between treatment groups were assessed using the extra sum-of-squares *F*-test. Statistical significance was defined as *P* < 0.05.

## 3 Results

### 3.1 Determination of the optimal ME concentration for preserving structural integrity

**Figure 1:**
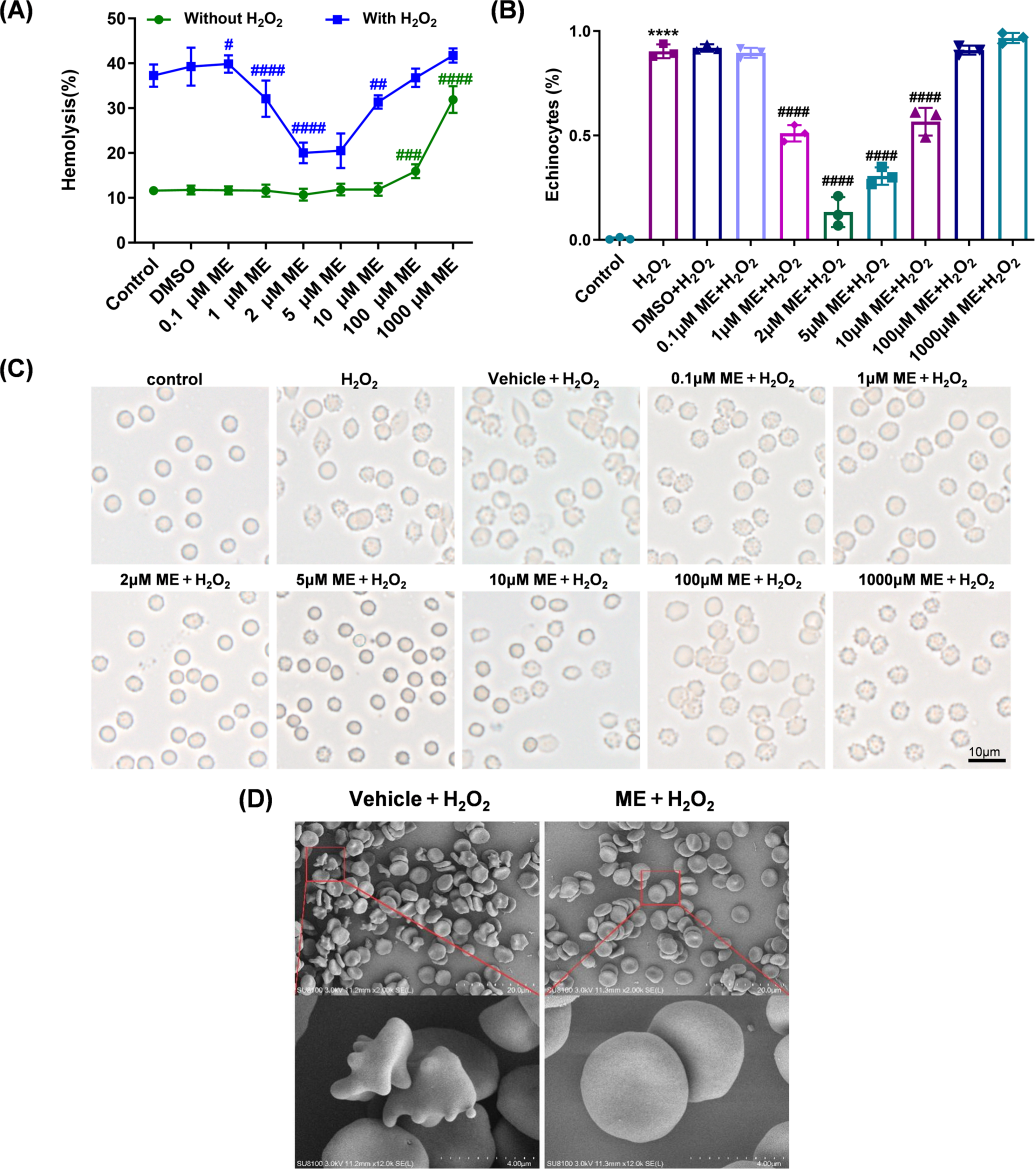
Determination of the optimal methyl eugenol (ME) concentration for preserving structural resilience. Erythrocytes were coincubated with dimethyl sulfoxide (DMSO) or varying concentrations of ME (0.1–1000 µM) and 300 µM H_2_O_2_ for 24 h. **(A)** Hemolysis rate. **(B)** Representative light microscopy images showing cell morphology alterations (400× magnification; scale bar = 10 µm). **(C)** Quantitative analysis of echinocytes presented as a jittered bar plot. **(D)** Scanning electron microscopy images of erythrocyte ultrastructure. Data are expressed as mean ± SD from independent biological replicates (n = 6 for panel **(A)**, n = 3 for **(B–D)**. ****P< 0.0001 *vs.* control group; ^#^*P* < 0.05, ^##^*P* < 0.01, ^###^*P* < 0.001, ^####^*P<* 0.0001 *vs.* Vehicle + H_2_O_2_ group.

To evaluate the protective potential of ME, erythrocytes were first exposed to an exogenous oxidative challenge using 300 µM H_2_O_2_, a concentration previously validated to induce progressive membrane deterioration without causing immediate or extensive hemolysis (Remigante et al., 2021, 2022b). ME was initially screened across a broad concentration range (0.1–1000 µM) to define its therapeutic window. Under basal conditions, ME exhibited high biocompatibility at concentrations up to 10 µM. However, suprapharmacological concentrations (100–1000 µM) elicited a significant propensity toward hemolysis, thereby defining the upper limits of safe use (**Figure 1A**).

Under H_2_O_2_ challenge, treatment with low-dose ME significantly attenuated hemolytic damage. Maximal efficacy was observed at 2 µM, where the hemolysis rate was reduced by approximately 50% compared with the Vehicle + H_2_O_2_ group (20.03% ± 2.30% vs. 39.27% ± 4.23%; *P<* 0.0001; **Figure 1A**). This functional preservation of erythrocyte integrity was corroborated by morphological analyses using light microscopy and high-resolution SEM. These analyses revealed that exposure to H_2_O_2_ triggered an extensive discocyte-to-echinocyte transition, resulting in a spiculated echinocytic phenotype (**Figures 1B–D**). Conversely, treatment with ME at 2 µM effectively prevented this transformation, maintaining the majority of erythrocytes in a smooth, biconcave discocytic morphology (**Figures 1C, D**).

Finally, cell-free assays revealed that ME does not directly react with H_2_O_2_ in solution, as no significant difference was observed compared with controls (*P* > 0.05; **Supplementary Figure 1**). This finding excludes nonspecific chemical quenching and suggests that the protective effects of ME are mediated through intracellular biotransformation or direct structural fortification of the membrane scaffold.

### 3.2 ME restores redox homeostasis and inhibits oxidative stress–induced eryptosis

**Figure 2:**
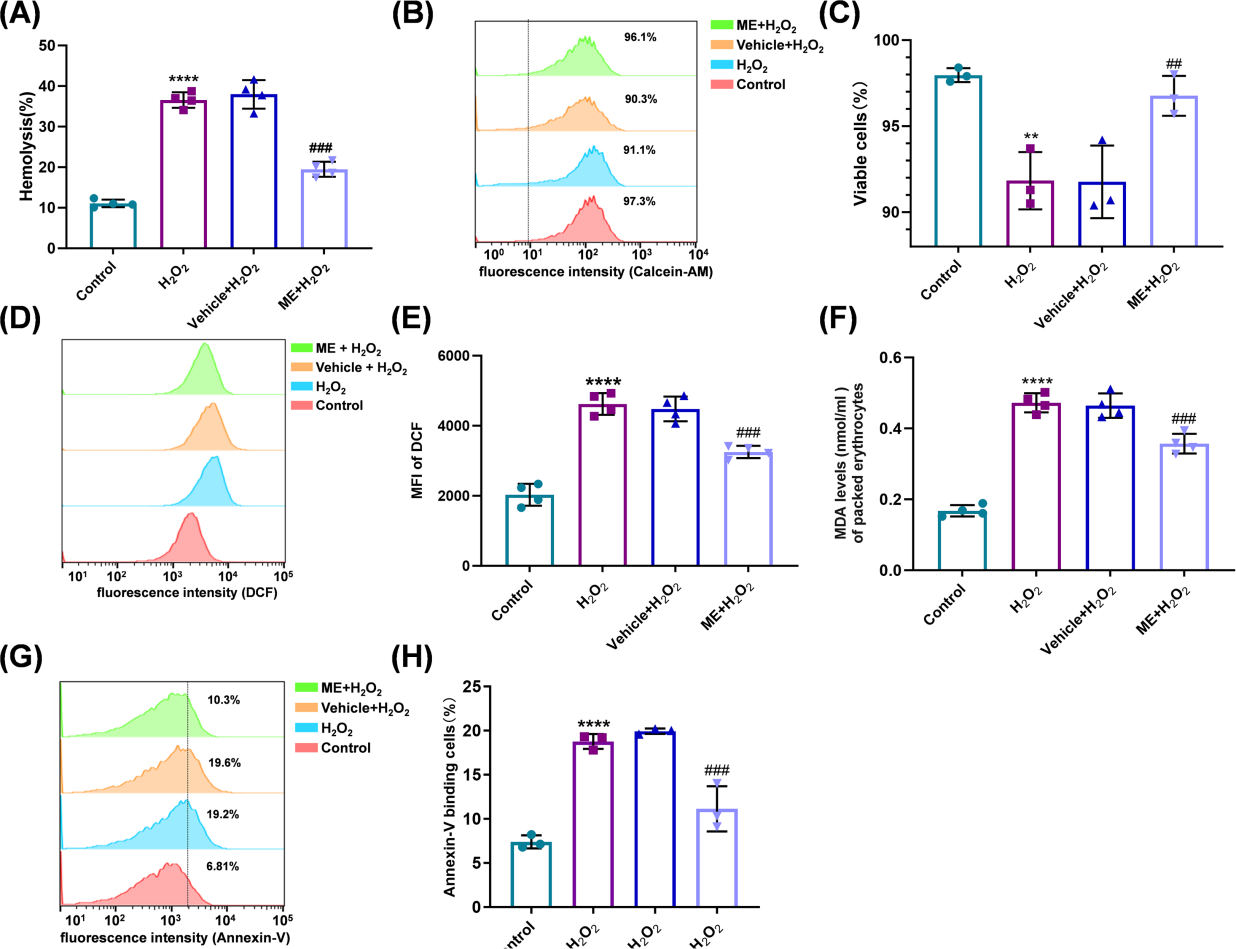
ME restores redox homeostasis and inhibits oxidative-induced eryptosis. Erythrocytes were coincubated with Vehicle or 2 µM ME and 300 µM H_2_O_2_ for 24 h. **(A)** Hemolysis rate. **(B)** Representative staggered histograms indicating cellular metabolic activity as determined using calcein-AM staining. **(C)** Percentage of metabolically active viable cells derived from calcein-AM staining analysis. **(D)** Representative histograms of intracellular reactive oxygen species (ROS) levels determined by 2’,7’-dichlorodihydrofluorescein diacetate (DCFH-DA). **(E)** Mean fluorescence intensity (MFI) of DCF across treatment groups. **(F)** Malondialdehyde (MDA) levels (nmol/mL of packed erythrocytes) indicate lipid peroxidation. **(G)** Representative staggered histograms indicating phosphatidylserine (PS) externalization using Annexin V staining. **(H)** Percentage of Annexin V^+^ cells. Data are expressed as mean ± SD from independent biological replicates [n = 4 for **(A)**, n = 3 for **(B–H)**]. **P< 0.01, ****P < 0.0001 *vs.* control group; ^#^*P* < 0.05, ^##^*P* < 0.01, ^###^*P* < 0.001, ^####^*P* < 0.0001 *vs.* Vehicle + H_2_O_2_ group.

Having identified 2 µM as the optimal structurally stabilizing concentration, we first conducted a focused phenotypic validation across the four core experimental groups. As shown in **Figure 2A**, treatment with ME (2 µM) effectively mitigated the pronounced increase in hemolysis induced by H_2_O_2_ exposure. We next examined whether this functional preservation was associated with the restoration of intracellular redox balance and the inhibition of eryptosis. This protective effect was further supported by flow cytometric analysis using calcein-AM staining (**Figure 2B**), which showed that treatment with ME markedly rescued the metabolically active viable cell population from the significant depletion observed in the Vehicle + H_2_O_2_ group (*P* = 0.0065; **Figure 2C**).

Given that membrane destabilization is driven by excessive oxidative burden, intracellular ROS levels and lipid peroxidation were subsequently quantified. Flow cytometric analysis using the DCFH-DA probe revealed that H_2_O_2_ exposure triggered a massive surge in ROS production (**Figure 2D**). ME treatment substantially attenuated this oxidative surge, shifting the MFI toward basal levels (*P* < 0.0001; **Figure 2E**). This antioxidant effect translated directly to the prevention of lipid deterioration, as evidenced by a marked suppression of elevated MDA levels, a definitive marker of oxidative lipid damage (0.357 ± 0.028 vs. 0.464 ± 0.034 nmol/mL in the Vehicle + H_2_O_2_ group; *P* = 0.0002; **Figure 2F**).

Finally, we assessed whether ME modulates eryptosis, the programmed clearance pathway characterized by PS externalization, using Annexin V–binding assays. H_2_O_2_ exposure resulted in more than a twofold increase in the PS^+^ cell population (**Figure 2G**). Reflecting the re-establishment of redox homeostasis, cotreatment with ME significantly attenuated this pro-eryptotic shift (*P* < 0.0001; **Figure 2H**). Collectively, these findings indicate that ME is an effective stabilizing agent, protecting erythrocytes from oxidative stress–induced membrane destabilization and premature clearance.

### 3.3 ME suppresses pathological Ca^2+^ influx and subsequent calpain activation

**Figure 3:**
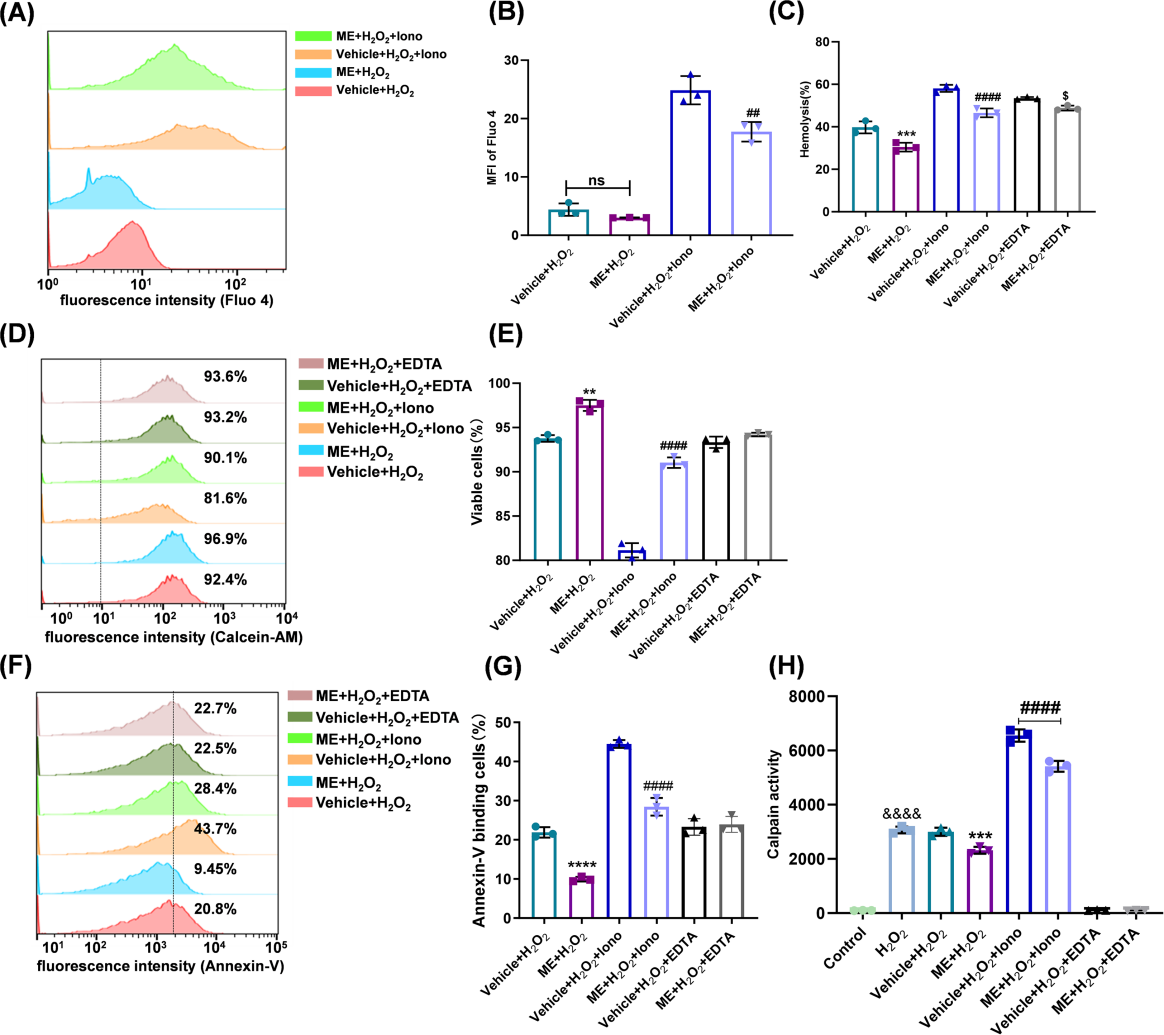
ME Suppresses Pathological Ca^2+^ Influx and Subsequent Calpain Activation. Erythrocytes were coincubated with DMSO or 2 µM ME and 300 µM H_2_O_2_ for 24 h. Where indicated, ionomycin (1 µM) or EDTA (1 mM) was added to the solution. **(A)** Representative staggered histograms of intracellular Ca^2+^ levels determined by Fluo-4 staining. **(B)** MFI of Fluo-4 across treatment groups. **(C)** Hemolysis rate. **(D)** Representative staggered histograms indicating cellular metabolic activity using calcein-AM. **(E)** Percentage of metabolically active viable cells derived from calcein-AM analysis. **(F)** Representative flow cytometry plots for PS externalization using Annexin V. **(G)** Percentage of Annexin V^+^ cells. **(H)** Calpain activity measured by fluorescence ratio (505/400 nm). Data are expressed as mean ± SD from independent biological replicates (n = 3). *P < 0.05, **P < 0.01, ***P< 0.001, ****P < 0.0001 *vs.* Vehicle + H_2_O_2_ group; ^##^*P* < 0.01, ^####^*P* < 0.0001 *vs.* Vehicle + H_2_O_2_ + ionomycin group; ^$^*P* < 0.05 vs. Vehicle + H_2_O_2_ + EDTA group; ^&&&&^*P* < 0.0001 *vs.* control group.

We next investigated the impact of ME on the Ca^2+^-calpain axis, a critical pathway initiating eryptosis. Under basal H_2_O_2_ insult, ME treatment resulted in a modest reduction in intracellular Ca^2+^ concentration ([Ca^2+^]_i_), but the regulatory potential of ME became markedly pronounced when erythrocytes were exposed to ionomycin to induce acute Ca^2+^ influx. Under the experimentally amplified ionic dysregulation of ionomycin, ME treatment significantly suppressed the pathological increase in [Ca^2+^]_i_ (24.87 ± 2.419 vs. 17.73 ± 1.677; *P* < 0.001; **Figures 3A, B**).

Paralleling this Ca^2+^ modulation, ME mitigated ionomycin-induced physiological damage. Specifically, treatment reduced hemolysis (**Figure 3C**), markedly suppressed pro-eryptotic PS externalization (**Figures 3D, E**), and rescued cellular viability (**Figures 3F, G**). To further confirm the causal role of extracellular Ca^2+^ influx, extracellular Ca^2+^ was chelated using EDTA. EDTA treatment alone reduced cellular injury to levels comparable to those observed in ME-treated groups, and under these Ca^2+^-depleted conditions, the additional protective effect of ME was largely diminished (**Figures 3C–G**).

To determine whether this regulation of Ca^2+^ influx translates to downstream enzymatic inhibition, we assessed the activity of calpain, the key effector protease. ME effectively mitigated the significant calpain activation elicited by H_2_O_2_ exposure (*P<* 0.001). This suppressive effect persisted even under robust ionomycin stimulation, where ME significantly attenuated enzymatic activity (*P<* 0.0001). Control experiments with EDTA abolished activity across all groups, confirming the strict Ca^2+^ dependency of the enzyme (**Figure 3H**). Collectively, these data demonstrate that ME inhibits calpain activation by restricting upstream Ca^2+^ influx.

### 3.4 ME directly stabilizes the cytoplasmic domain of Band 3 against proteolytic cleavage

**Figure 4:**
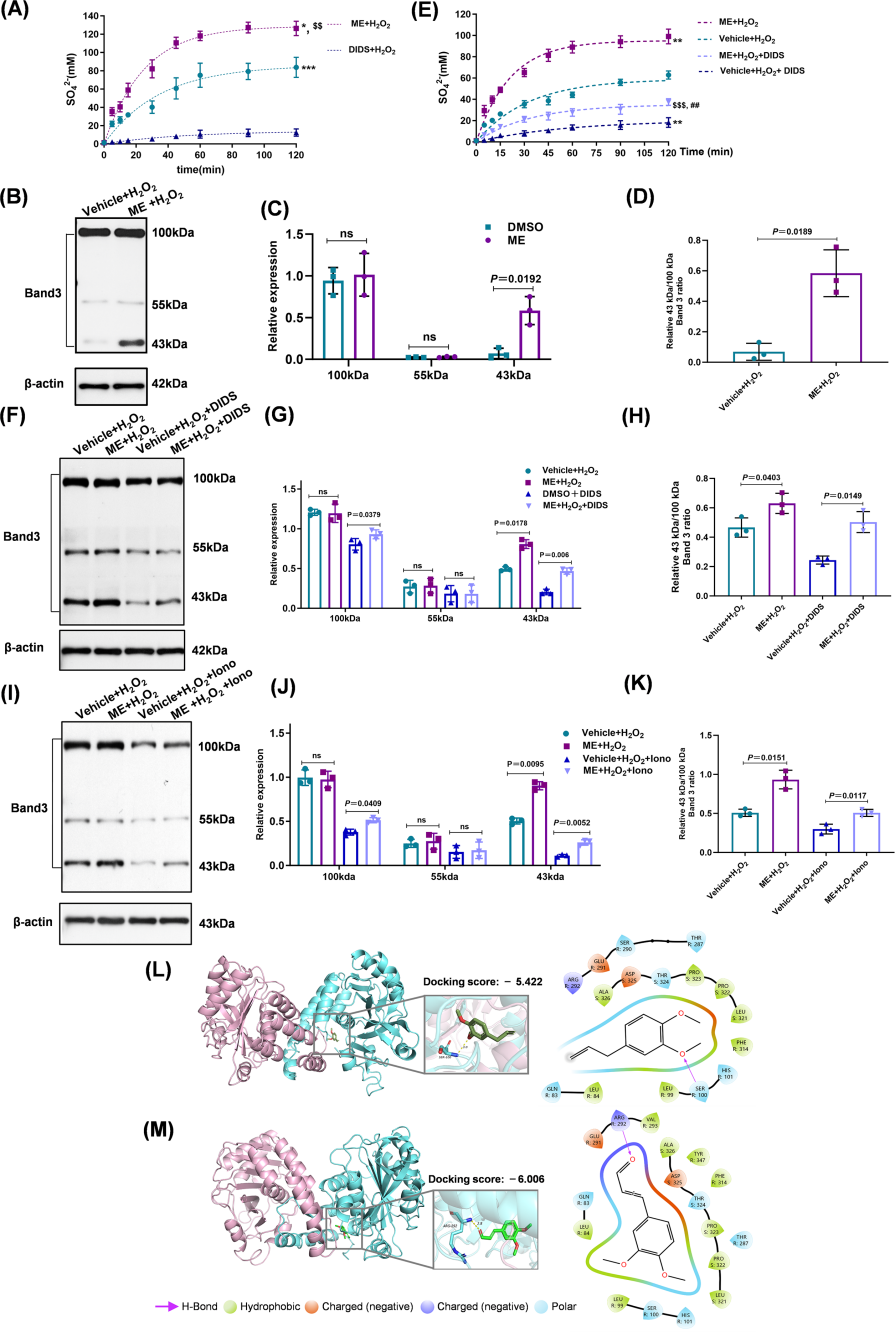
ME directly stabilizes the cytoplasmic domain of band 3 against proteolytic cleavage. Erythrocytes were preincubated with 4,4′-diisothiocyanatostilbene-2,2′-disulfonate (DIDS; 10 µM) for 30 min, or preincubated with ionomycin (1 µM), where indicated, and coincubated with DMSO or 2 µM ME and 300 µM H_2_O_2_ for 24 h. **(A)** SO_4_^2−^ uptake kinetics measured by turbidimetry to assess Band 3 transport function. **(B, C)** Western blot and quantification of full-length (100 kDa), transmembrane (55 kDa), and cytoplasmic (43 kDa) Band 3 fragments. Loading control: β-actin. **(D)** Relative 43 kDa/100 kDa Band 3 ratio representing the preservation of the cytoplasmic domain (structural stabilization index). **(E)** SO_4_^2−^ uptake rate in erythrocytes treated with DIDS. **(F, G)** Western blot and quantification of Band 3 protein fragments in the presence of DIDS. Loading control: β-actin. **(H)** Relative 43 kDa/100 kDa Band 3 ratio representing the structural stabilization index in the presence of DIDS. **(I, J)** Western blot and quantification of Band 3 protein fragments in the presence of ionomycin. Loading control: β-actin. **(K)** Relative 43 kDa/100 kDa Band 3 ratio representing the structural stabilization index in the presence of ionomycin. **(L, M)** Molecular docking simulations depicting 3D ribbon structures (left), close-up binding pockets (middle), and two-dimensional interaction diagrams (right). **(L)** Docking complex of ME with Band 3. **(M)** Docking complex of the ME aldehyde derivative with Band 3. Data are expressed as mean ± SD from independent biological replicates (n = 3). ** *P* < 0.01, ***P< 0.001 *vs.* Vehicle + H_2_O_2_ group; ^#^*P* < 0.05, ^##^P< 0.01 *vs.* ME + H_2_O_2_ group, respectively. ^$$^*P* < 0.01 *vs.* Vehicle+ H_2_O_2_ + DIDS group.

Since calpain activation targets the erythrocyte membrane scaffold, we examined whether ME protects Band 3 from proteolytic cleavage and maintains its structural integrity (Romero et al., 2013). First, we assessed anion exchange kinetics as a functional readout. ME treatment significantly enhanced transport velocity compared with untreated controls (**Figure 4A**). Notably, the ME + H_2_O_2_ group achieved transport equilibrium within 25 min, whereas the Vehicle + H_2_O_2_ group required 35 min (*P* < 0.01; **Supplementary Table 1**), indicating that ME maintains the intrinsic functional capacity of Band 3 under oxidative stress.

Band 3 is a 100 kDa integral membrane protein comprising a 55 kDa transmembrane domain and a 43 kDa cytoplasmic domain (Low, 1986). We monitored the structural stability of these regions under oxidative stress using western blotting (**Figure 4B**). The abundance of the 100 kDa full-length and the 55 kDa transmembrane domain remained comparable across treatment groups (*P* > 0.05; **Figure 4C**). However, ME treatment selectively preserved the 43 kDa cytoplasmic domain (0.87 ± 0.08 vs. 0.67 ± 0.05; *P* = 0.0192; **Figure 4C**). To further quantify this effect, the 43/100 kDa ratio was calculated as an index of structural stabilization. The ME + H_2_O_2_ group showed a significantly higher ratio than the Vehicle + H_2_O_2_ group (*P* = 0.0189; **Figure 4D**). Since the abundance of the full-length protein was not significantly altered (*P* > 0.05), these data indicate that ME acts primarily by intercepting the secondary proteolytic breakdown of the 43 kDa fragment, effectively ‘trapping’ this intermediate in a stable conformation rather than preventing the initial cleavage event.

To determine if this protection depended on anion transport function, we utilized the specific inhibitor DIDS. Although DIDS significantly inhibited anion transport kinetics (**Figure 4E**, **Supplementary Table 2**), ME retained its capacity to stabilize the Band 3 protein structure. Even in the presence of DIDS, ME maintained significantly higher levels of both the 100 and 43 kDa bands compared with the Vehicle + H_2_O_2_ + DIDS group (**Figures 4F, G**), suggesting a structural stabilizing mechanism independent of anion transport inhibition. This structural preservation was quantitatively confirmed by ratio analysis, which showed that the 43/100 kDa ratio remained significantly higher in the ME + H_2_O_2_ + DIDS group than in the Vehicle + H_2_O_2_ + DIDS group (*P* = 0.0149; **Figure 4H**).

To evaluate the physiological relevance of this structural rescue, we assessed cellular outcomes in the presence of DIDS. While DIDS-mediated functional blockade partially weakened the protection against hemolysis (**Supplementary Figure 3A**), ME maintained a robust survival advantage. Specifically, ME preserved cell viability (**Supplementary Figures 3B, C**) and effectively inhibited PS externalization (**Supplementary Figures 3D, E**) compared with DIDS controls. These data suggest that by stabilizing the 43 kDa cytoplasmic domain, ME maintains a structural framework sufficient to support cellular survival even when anion transport is compromised.

The robustness of this stabilization was further tested under severe proteolytic stress. Ionomycin led to a substantial depletion in all Band 3 components, thereby accentuating the protective effect of ME. Under these severe conditions, ME cotreatment significantly preserved the integrity of both the 100 and 43 kDa fragments (*P* = 0.0409 and 0.0052, respectively; **Figures 4I, J**). Furthermore, ME treatment significantly increased the 43/100 kDa ratio under both H_2_O_2_- and ionomycin-induced stress (*P* = 0.0151 and 0.0117, respectively; **Figure 4K**), confirming structural stabilization across different stress models.

To explore the potential molecular basis for these observations, we performed *in silico* docking simulations. The results suggested that the aldehyde derivative of ME exhibited a high binding affinity (docking score: −6.006 kcal/mol) and formed predicted stable hydrogen bonds with residue ARG292 within the 43 kDa cytoplasmic domain of Band 3 (**Figures 4L, M**; **Supplementary Figure 2**). This interaction was further predicted to occupy a hydrophobic pocket formed by VAL293 and ALA326 (**Figure 4M**), suggesting a potential physical interaction that may contribute to the observed resistance against proteolysis.

### 3.5 ME-mediated cytoprotection is independent of endogenous catalase activity

To determine whether the cytoprotective effects of ME depend on augmentation of endogenous antioxidant defenses, catalase activity was inhibited using the specific inhibitor 3-AT. As expected, catalase inhibition exacerbated H_2_O_2_-induced oxidative injury. However, ME conferred robust protection even in the absence of functional catalase activity.

**Figure 5:**
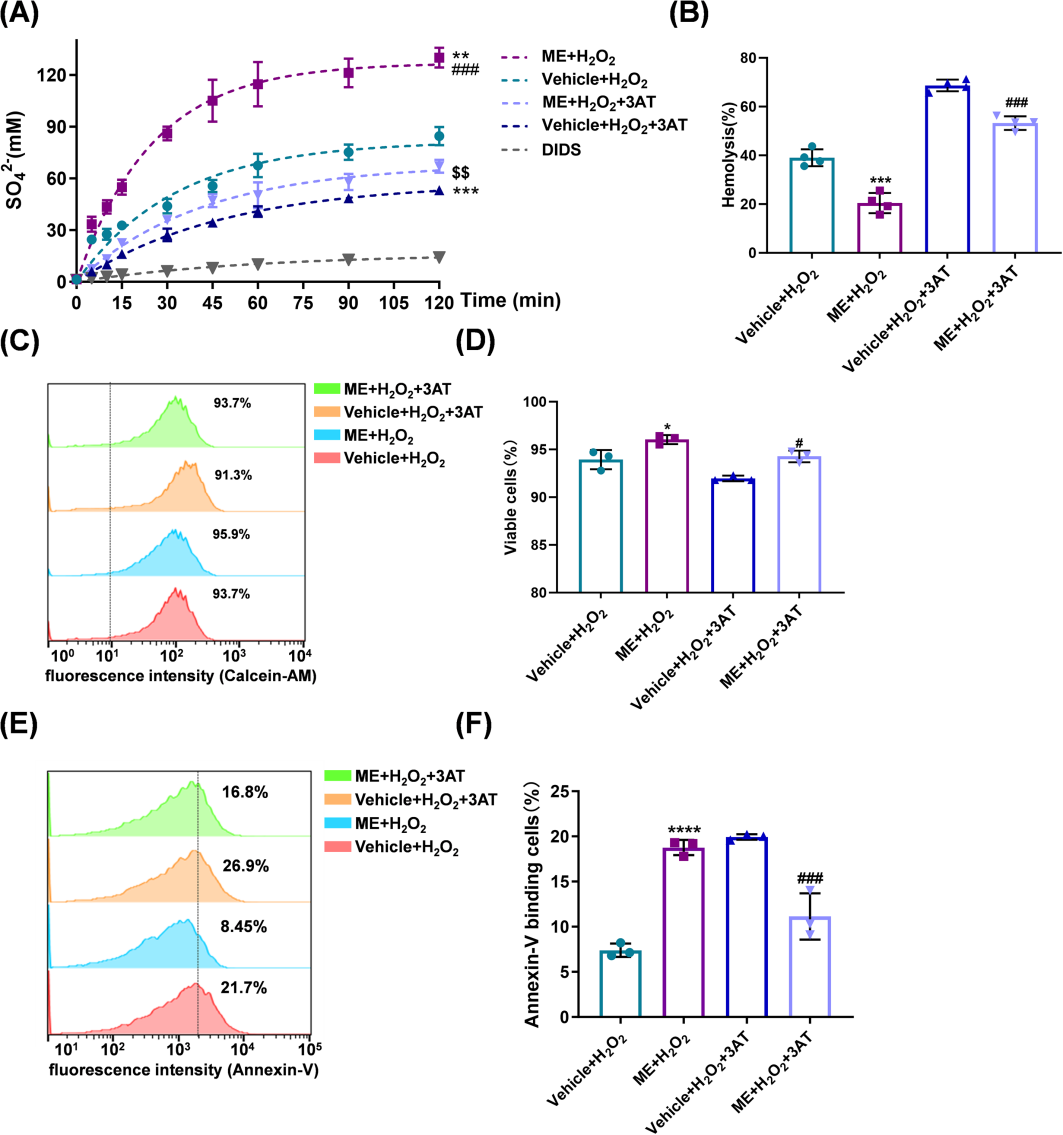
ME-mediated cytoprotection is independent of endogenous catalase activity. Erythrocytes were coincubated with DMSO or 2 µM ME and 300 µM H_2_O_2_ for 24 h. Where indicated, erythrocytes were pretreated with 1 mM 3-amino-1,2,4-triazole (3-AT) for 1 h. **(A)** SO_4_^2−^ uptake kinetics measured by turbidimetry to assess Band 3 transport function. **(B)** Hemolysis rate. **(C)** Representative staggered histograms indicating cellular metabolic activity using calcein-AM. **(D)** Percentage of metabolically active viable cells derived from calcein-AM analysis. **(E)** Representative staggered histograms showing PS externalization determined using Annexin V. **(F)** Percentage of Annexin V^+^ cells. Data are expressed as mean ± SD from independent biological replicates [n = 4 for **(A, B)**, n = 3 for **(C–F)**]. *P < 0.05, **P< 0.01, ***P< 0.001, ****P< 0.0001 *vs.* Vehicle + H_2_O_2_ group; ^#^*P* < 0.05, ^###^*P* < 0.001, ^####^*P<* 0.0001 *vs.* ME + H_2_O_2_ + 3-AT group; ^$$^*P* < 0.01 *vs.* Vehicle + H_2_O_2_ + 3-AT group.

Functional integrity of Band 3 was first evaluated by measuring SO_4_^2−^ uptake kinetics (**Figure 5A**). H_2_O_2_ exposure significantly suppressed SO_4_^2−^ uptake, an effect further intensified by the addition of 3-AT. Notably, ME treatment largely restored transport velocity under these compromised conditions, effectively mitigating the loss of Band 3–mediated anion exchange (**Figure 5A**; **Supplementary Table 3**). Hemolysis assays further supported these findings. Although catalase inhibition increased susceptibility to oxidative damage, ME consistently maintained hemolysis at significantly lower levels than those observed in corresponding vehicle-treated groups (**Figure 5B**).

The effects of ME on eryptotic markers were next evaluated using flow cytometry. Calcein-AM staining revealed that ME preserved membrane integrity and cellular viability even in the presence of 3-AT (**Figures 5C, D**). Furthermore, Annexin V–binding assays demonstrated that ME markedly attenuated the proportion of PS-exposed cells, even when endogenous catalase activity was pharmacologically silenced (**Figures 5E, F**). Collectively, these observations indicate that the cytoprotective capacity of ME is independent of catalase-mediated H_2_O_2_ scavenging. This suggests that ME operates through a distinct mechanistic pathway, likely involving direct radical neutralization or structural stabilization, to protect erythrocytes from oxidative disintegration.

### 3.6 ME ameliorates age-associated redox decline in a human donor cohort

**Figure 6:**
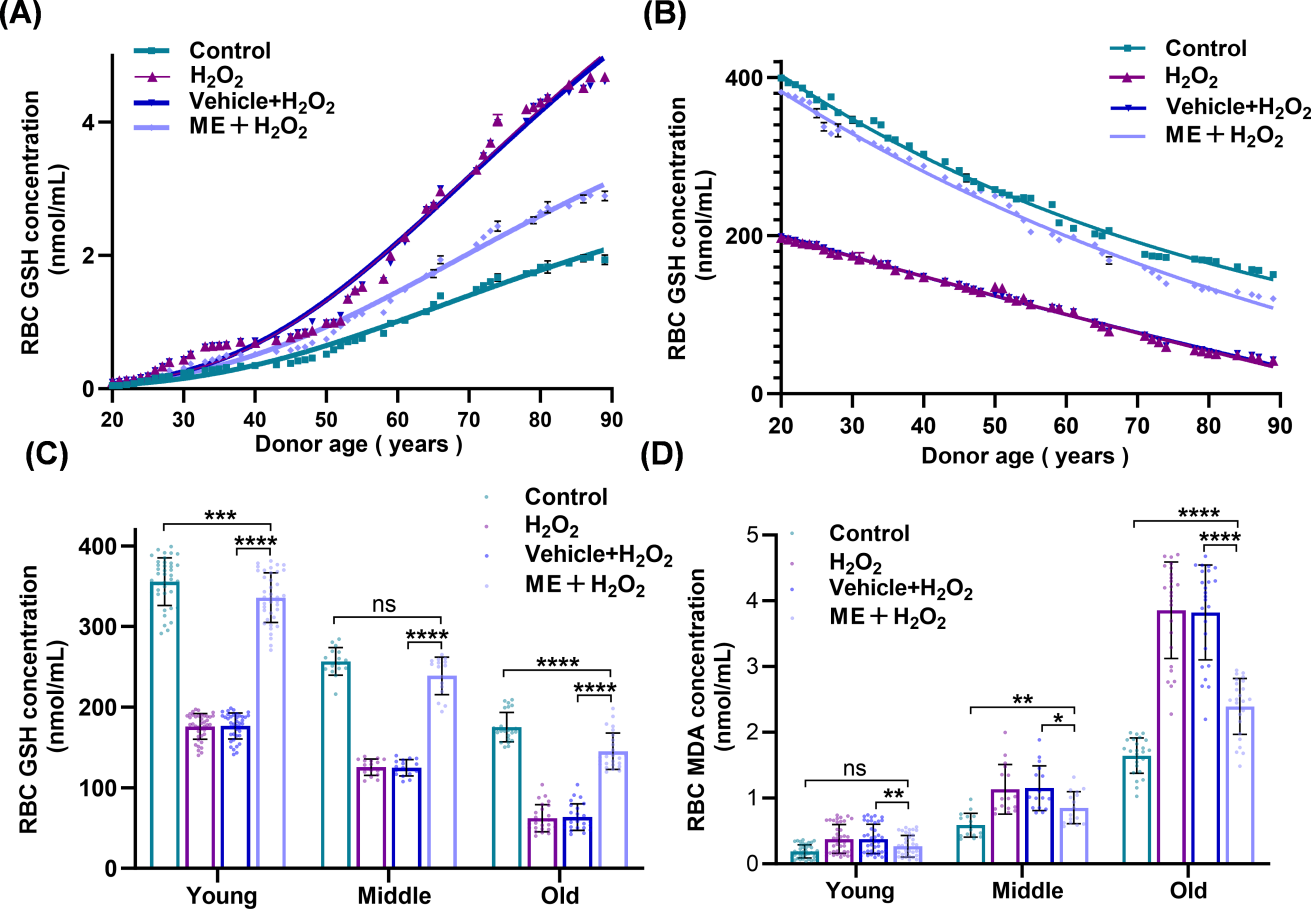
ME Ameliorates Age-Associated Redox Decline in a Human Donor Cohort. **(A, B)** Kinetic trajectories of erythrocyte redox markers across the donor cohort (N = 81). Solid lines denote best-fit regression curves for **(A)** Glutathione (GSH) concentration (one-phase exponential decay) and **(B)** MDA levels (Gompertz growth regression). **(C, D)** Age-stratified analysis of **(C)** GSH and **(D)** MDA concentrations according to the following subgroups: young (20–44 years, n = 40), middle (45–59 years, n = 16), and old (60–90 years, n = 25). Data are expressed as mean ± SD **(C, D)** or regression best-fits **(A, B)**. *P< 0.05, **P< 0.01, ***P< 0.001, ****P< 0.0001; ns, not significant.

To evaluate the effects of ME across a broader physiological spectrum, regression modeling was performed using erythrocytes from healthy donors of varying ages. One-phase exponential decay analysis revealed that GSH levels in control erythrocytes steadily declined with increasing donor age (Y_0_ = 541.4 nmol/mL), corresponding to an age-related half-life of 47.14 years (**Figure 6A**; **Table 1**). Although acute H_2_O_2_ exposure significantly depleted antioxidant reserves, ME treatment restored baseline GSH levels to 508.1 nmol/mL and extended the age-dependent half-life to 64.14 years. Notably, extra sum-of-squares *F*-test analysis revealed that ME significantly altered the overall age-dependent GSH kinetic profile compared with the Vehicle + H_2_O_2_ group (*P* < 0.0001; **Table 1**). Stratified comparisons using Tukey’s *post hoc* analysis further confirmed that ME conferred significant protection across all age cohorts (*P* < 0.0001; **Figure 6C**).

**Table 1:** Core parameters of age-dependent erythrocyte GSH concentration changes (derived from one-phase exponential decay fitting).

Group	Baseline (Y_0_, nmol/mL) (95%CI)	Annual decay rate (K) (95%CI)	Age half-life (years) (95%CI)	R²	Sy.x	n	P
Control	541.4 (528.5~555.1)	0.01470 (0.01277~0.01666)	47.14 (41.61~54.29)	0.9950	5.992	81	–
H_2_O_2_	251.8 (245.3~258.7)	0.002353 (0.0002229~0.004497)	294.5 (154.1~3110)	0.9933	4.333	81	*P* < 0.0001^a^
DMSO+H_2_O_2_	256.0 (250.5~261.9)	0.003823 (0.002053~0.005603)	181.3 (123.7~337.7)	0.9954	3.558	81	*P*>0.05^b^
ME+H_2_O_2_	508.1 (492.7.8~524.5)	0.01081 (0.008385~0.01326)	64.14 (52.29~82.67)	0.9919	8.046	81	*P* < 0.0001^c^

(1) Model formula: ${\text{Y}}_{0}\times e^{\left(-\text{K}\times \text{X}\right)}$ (Y: erythrocyte GSH concentration, Y_0_: baseline GSH in young donors, K: age-dependent annual decay rate, X: donor age). Age half-life = (ln(2)/K) (age for GSH reduction to 50% of baseline). (2) All groups showed high fitting reliability (R² > 0.99). (3) Negative plateau values were not listed for no biological meaning. (4) For the H_2_O_2_ group, the extremely wide upper limit of age half-life (3110 years) stems from the markedly slowed age-dependent GSH decay rate and relatively high data dispersion post H_2_O_2_ exposure, impairing estimation precision of the decay rate (K); this value has no practical biological significance in the 20–90-year-old donor cohort. 4. a vs. Control group, b: vs. H_2_O_2_ group, c: vs. DMSO+H_2_O_2_ group.

Gompertz growth regression was used to model the accumulation kinetics of MDA. In the control group, the maximal accumulation plateau (Y_M_) was 3.423, which increased sharply to 8.610 under oxidative stress (**Figure 6B**; **Table 2**). ME treatment substantially reshaped this age-dependent trajectory. Extra sum-of-squares *F*-test analysis confirmed a significant alteration in the fitting parameters (*P<* 0.0001 vs. Vehicle + H_2_O_2_; **Table 2**), reducing the Y_M_ value to 5.258, corresponding to a 38.9% decrease in the theoretical maximum lipid damage. Stratified *post hoc* comparisons further validated that ME effectively mitigated MDA accumulation across all age cohorts (**Figure 6D**).

**Table 2:** Core parameters of age-dependent changes in erythrocyte MDA levels across groups (Gompertz growth regression).

Group	Y_M_ (plateau maximum) (95% CI)	Y_0_ (initial baseline) (95% CI)	K (growth rate constant) (95% CI)	1/K (characteristic time, years) (95% CI)	R²	Sy.x	n	P
Control	3.423 (2.994–4.051)	0.001317 (0.0003387–0.003684)	0.03106 (0.02688–0.03553)	32.19 (28.15–37.20)	0.9874	0.07595	81	–
H_2_O_2_	8.744 (7.322–11.04)	0.0008940 (0.00007512–0.004496)	0.03162 (0.02623–0.03771)	31.63 (26.52–38.13)	0.9834	0.2100	81	<0.0001^a^
H_2_O_2_+DMSO	8.610 (7.235–10.82)	0.001078 (0.0001080–0.004995)	0.03143 (0.02616–0.03732)	31.82 (26.79–38.23)	0.9833	0.2082	81	>0.05^b^
H_2_O_2_+ME	5.258 (4.610–6.191)	0.002264 (0.0007205–0.005567)	0.02997 (0.02623–0.03392)	33.37 (29.48–38.13)	0.9896	0.1010	81	<0.0001^c^

(1) Model formula: $Y=Y_{M}\times e^{-e^{\left(K\times \left(t_{0}-X\right)+1\right)}}$ (Y: erythrocyte MDA level, X: donor age, Y_M_: maximum plateau value of MDA accumulation; Y_0_: initial MDA level in young donors; K: rate constant of age-related MDA growth; 1/K: time required to approach the plateau). (2) Statistical analysis: extra sum-of-squares *F*-test; P< 0.05 was considered statistically significant. a: vs. Control group, b: vs. H_2_O_2_ group, c: vs. DMSO+H_2_O_2_ group. (3) All groups met model constraints (Y_0_ > 0, K > 0), with high fitting reliability (R² ≥ 0.9836). n: number of samples (donors); Sy.x: standard error of the estimate.

**Figure 7:**
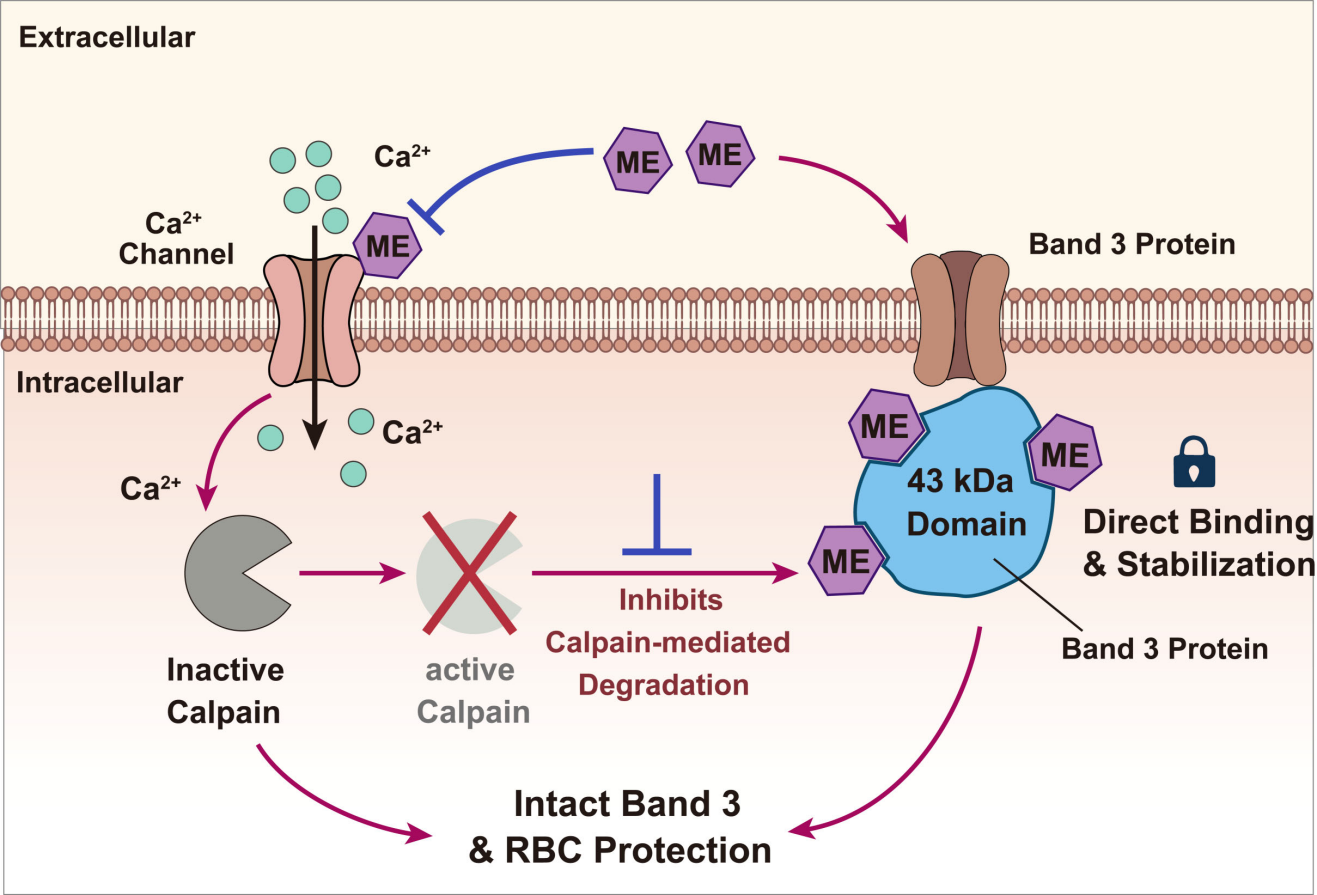
ME preserves erythrocyte quality during aging by targeting oxidative and proteolytic damage. The mechanism involves direct stabilization of the membrane skeleton: ME traps the 43 kDa cytoplasmic domain of Band 3, blocking its terminal degradation via inhibition of the Ca^2+^–calpain proteolytic axis.

## 4 Discussion

The susceptibility of human erythrocytes to cumulative oxidative damage is a key contributor to storage-related quality decline and may underlie the variable efficacy of blood from older donors (van Der Meer and Klei, 2023). Our data establish that ME confers cytoprotection through a defined mechanism that couples upstream inhibition of calcium overload with downstream structural stabilization of Band 3, distinct from simple radical scavenging (**Figure 7**).

### 4.1 Dose-dependent efficacy and metabolic fate

Dose–response analysis revealed a narrow therapeutic window for ME, with 2 µM identified as the optimal concentration for preserving cellular integrity. While the 300 µM H_2_O_2_ utilized to evaluate this efficacy exceeds physiological levels, it serves as a validated “time-compression” tool that effectively recapitulates chronic oxidative aging phenotypes within an acute experimental window (Shan et al., 2016; Remigante et al., 2022a). At this dosage, ME effectively attenuated morphological abnormalities, reduced hemolysis, and restored redox balance by reducing MDA levels (**Figures 1**, **2**). Notably, ME inhibited the defining events of eryptosis, including PS externalization and the pathological elevation of intracellular Ca^2+^ levels (**Figure 3**). In contrast, the transition to hemolytic toxicity observed at supratherapeutic concentrations (≥100 µM) suggests a biphasic pharmacological profile. This likely reflects the saturation of specific metabolic pathways within erythrocytes. While low doses favor the generation of stabilizing intermediates, high concentrations may overwhelm enzymatic capacity, potentially diverting ME toward the formation of reactive electrophiles (Orrico et al., 2023; Liu et al., 2022). Although future targeted metabolomic profiling is required to definitively map this boundary, our findings delineate a critical low-dose window that provides a necessary framework for potential therapeutic applications.

### 4.2 Structural stabilization of the Band 3 scaffold

A central finding of this study is that ME preserves the functional and structural integrity of Band 3 (Reithmeier et al., 2016), the central anchor of the erythrocyte membrane. We provide direct experimental evidence that ME treatment selectively preserves the 43-kDa cytoplasmic fragment (**Figures 4C, J**). This phenomenon, quantified using the 43/100-kDa ratio as an index of structural stabilization (**Figures 4D, K**), indicates that ME prevents the proteolytic disintegration of the membrane–cytoskeleton interface.

To rationalize this domain-specific protection, molecular docking simulations generated a structural hypothesis: ME metabolites exhibit high computational affinity for the ARG292 residue within the Band 3 cytoplasmic domain. This interaction may introduce steric hindrance or conformational stabilization, thereby protecting Band 3 from terminal proteolysis. The functional independence of this structural stabilization was confirmed using the Band 3 inhibitor DIDS. Although DIDS blocked anion transport and partially attenuated the anti-hemolytic efficacy of ME (**Supplementary Figure 3A**), it failed to abolish the survival benefit (**Supplementary Figures 3B–E**) or the preservation of the 43-kDa fragment (**Figures 4F–H**). These findings indicate that ME stabilizes the cytoplasmic domain through a mechanism distinct from the transmembrane anion transport function inhibited by DIDS (Passow, 2005).

### 4.3 Regulation of the Ca^2+^–calpain axis

In addition to this structural fortification, we identified a critical upstream regulatory role for ME. ME-mediated modulation of intracellular Ca^2+^ was stress-dependent: ME caused only a modest reduction under moderate H_2_O_2_ stress but significantly blunted the catastrophic Ca^2+^ surge induced by ionomycin (**Figure 3B**). This pattern suggests that ME does not function as a nonspecific Ca^2+^ chelator, which could disrupt basal signaling, but instead acts as a homeostatic stabilizer that selectively counteracts pathological Ca^2+^ overload. Critically, suppression of this initiating signal directly translated into the attenuation of calpain activity (**Figure 3H**), which in turn markedly reduced the cleavage of the Band 3 cytoplasmic domain (**Figures 4I–K**). The protective efficacy of ME was diminished when extracellular Ca^2+^ was chelated with EDTA, highlighting the requirement for modulating extracellular Ca^2+^ influx to achieve the full cytoprotective effect of ME (**Figures 3C–G**). The precise molecular triggers and targets underlying ME-mediated inhibition of Ca^2+^ influx remain to be identified. Given its lipophilic properties, a plausible mechanism is that ME or its active metabolites interact with plasma membrane components, such as ion channels or lipid raft domains, thereby modulating Ca^2+^ permeability (Cahalan et al., 2015; Föller et al., 2008). Identification of this upstream target will be essential for fully delineating the protective signaling cascade initiated by ME. Such membrane–protein interactions may represent the earliest event in the protective mechanism of ME, effectively decoupling oxidative stress from activation of the deleterious Ca^2+^–calpain–Band 3 axis.

### 4.4 Independent of enzymatic scavenging

Importantly, this coordinated mechanism operates independently of canonical antioxidant enzymes, as evidenced by the preservation of the protective effects of ME even in the presence of the catalase inhibitor 3-AT (**Figure 5**). These findings highlight that the cytoprotective action of ME is driven primarily by the maintenance of membrane–protein homeostasis and regulatory signaling rather than by enzymatic scavenging of oxidants.

### 4.5 Translational relevance: standardizing donor variability

Perhaps the most translationally relevant finding is the consistent efficacy of ME across a broad age range of human donors (20–90 years). Our data confirmed that erythrocytes from older donors were more susceptible to oxidative stress, evidenced by lower baseline GSH levels and higher MDA concentrations (**Figures 6A, B**). Using one-phase exponential decay analysis (n = 81), we showed that ME restored baseline GSH levels to 508.1 nmol/mL and significantly extended the age-dependent half-life from 47.14 to 64.14 years. Given the 120-day lifespan of erythrocytes, this projected half-life reflects systemic donor aging rather than cellular longevity. It demonstrates that older donors produce erythrocytes with lower baseline antioxidant defenses, a decline effectively mitigated by ME. Concurrently, Gompertz growth modeling showed that ME reduced the lipid peroxidation accumulation ceiling (Y_M_) from 8.610 to 5.258, corresponding to an approximate 39% reduction (**Figures 6C, D**). The high goodness-of-fit values (R^2^ ≥ 0.98) across the donor cohort underscore the robustness of the protective effect of ME, suggesting that ME effectively “standardizes” erythrocyte resilience and narrows the disparity in oxidative susceptibility between younger and older donors.

### 4.6 Limitations and future directions

While this study elucidates a novel protective mechanism, several avenues for future research remain. First, our current model employed an acute H_2_O_2_-induced oxidative challenge. Although effective for mechanistic dissection, the long-term effects of ME on erythrocytes under physiological storage conditions, particularly regarding critical functional parameters such as deformability, oxygen release kinetics, and vesiculation, remain to be determined. Second, although molecular docking generated a robust structural hypothesis for domain-specific protection, the lack of direct biophysical validation remains a limitation of this study. Future research must confirm this physical interaction using techniques such as surface plasmon resonance or cellular thermal shift assays. Third, the precise identity of the intracellularly active ME metabolite(s) in erythrocytes warrants clarification through targeted metabolomic analyses. Finally, these promising *ex vivo* findings justify further validation in animal models of transfusion or aging to precisely define the toxicological boundaries and assess the *in vivo* efficacy and circulatory survival of ME-treated erythrocytes.

## 5 Conclusion

In summary, ME preserves erythrocyte integrity through a coupled mechanism involving inhibition of the Ca^2+^–calpain axis and stabilization of Band 3. ME not only preserves the full-length protein under severe stress but also specifically intercepts terminal degradation, trapping the vital 43-kDa cytoplasmic domain in a stable intermediate state, thereby preventing structural collapse of the erythrocyte membrane. Regression analysis across donors (n = 81) confirmed that ME modulates redox kinetics by extending the effective half-life of GSH while lowering the maximal accumulation of MDA. Collectively, these findings indicate that ME represents a viable additive capable of enhancing and standardizing the quality and resilience of blood products across the human lifespan.

## Data Availability

The original contributions presented in the study are included in the article/**Supplementary Material**. Further inquiries can be directed to the corresponding authors.
