# Supplementary material for: Methyl eugenol attenuates age-associated oxidative fragility by coupling Ca2+-calpain inhibition with Band 3 stabilization in human erythrocytes
Source: Front Physiol. 2026 Apr 20;17:1796160. doi: 10.3389/fphys.2026.1796160 (PMC13135961; doi:10.3389/fphys.2026.1796160)
Supplement: Supplementary file 1 [file SupplementaryFile1.docx]

Supplementary Material

**Supplementary Figure 1.**

**
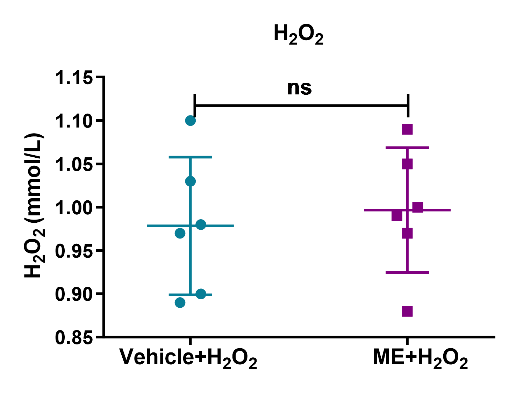
**

**Supplementary Figure 1. ME itself did not react with hydrogen peroxide in solution in vitro.** The concentration of hydrogen peroxide in the solution was detected after incubated 2μM ME with 300μM H_2_O_2_ for 24h in distilled water in 37℃ with slightly shaking. The experiment was repeated at least three times. ns: *P* > 0.05, *vs.* Vehicle+H_2_O_2_ group.

**Supplementary Figure 2.**


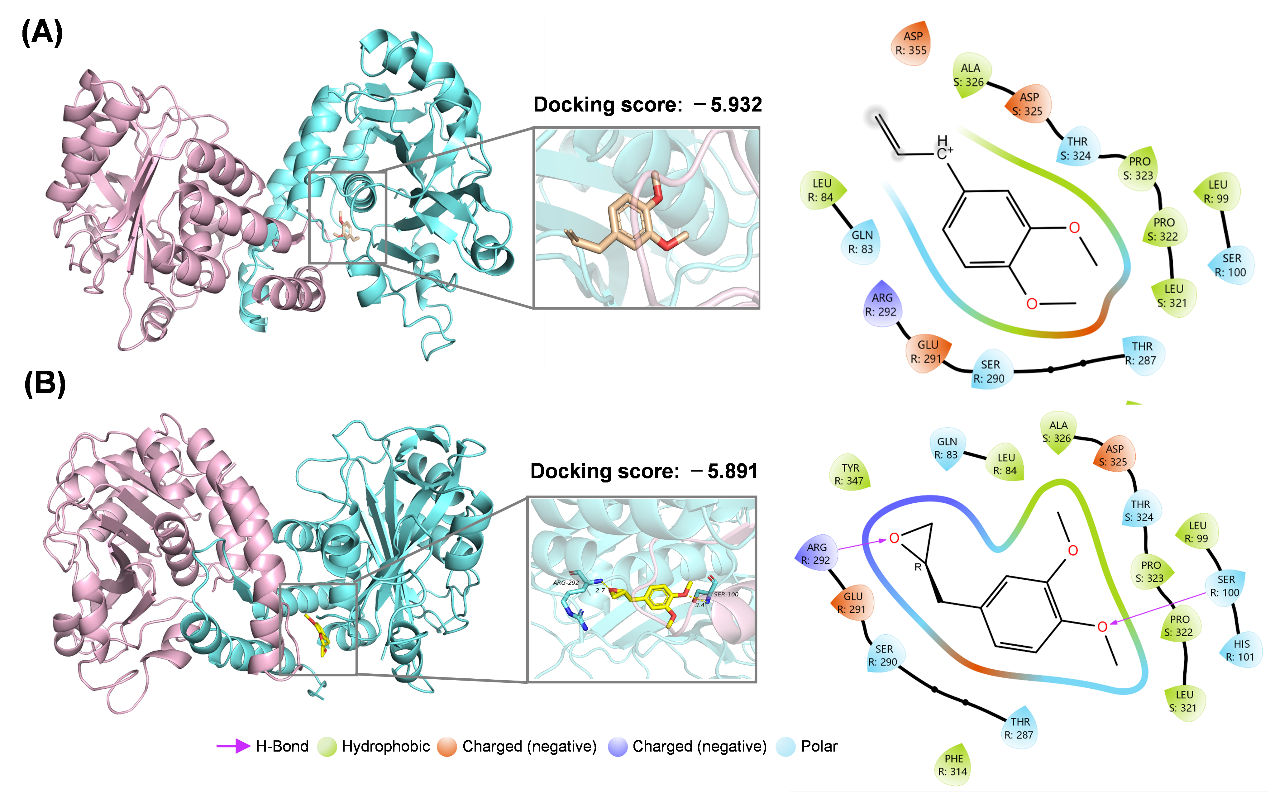


**Supplementary Figure 2. Molecular docking assays of ME’s epoxide derivative and cationic derivative with Band 3 protein.** The left panel depicts the 3D ribbon structure, the middle panel is a close-up view of the binding pocket, the right panel shows the 2D interaction diagram. (**A).** Docking complex of the epoxide derivative of ME (3,4-dimethoxy-cinnamaldehyde, green) with Band 3 (pink and cyan); (**B).** Docking complex of the cationic derivative of ME (1,2-Dimethoxy-4-(2-propen-1-ylium) benzene, tan) with Band 3 (pink and cyan).

**Supplementary Figure 3.
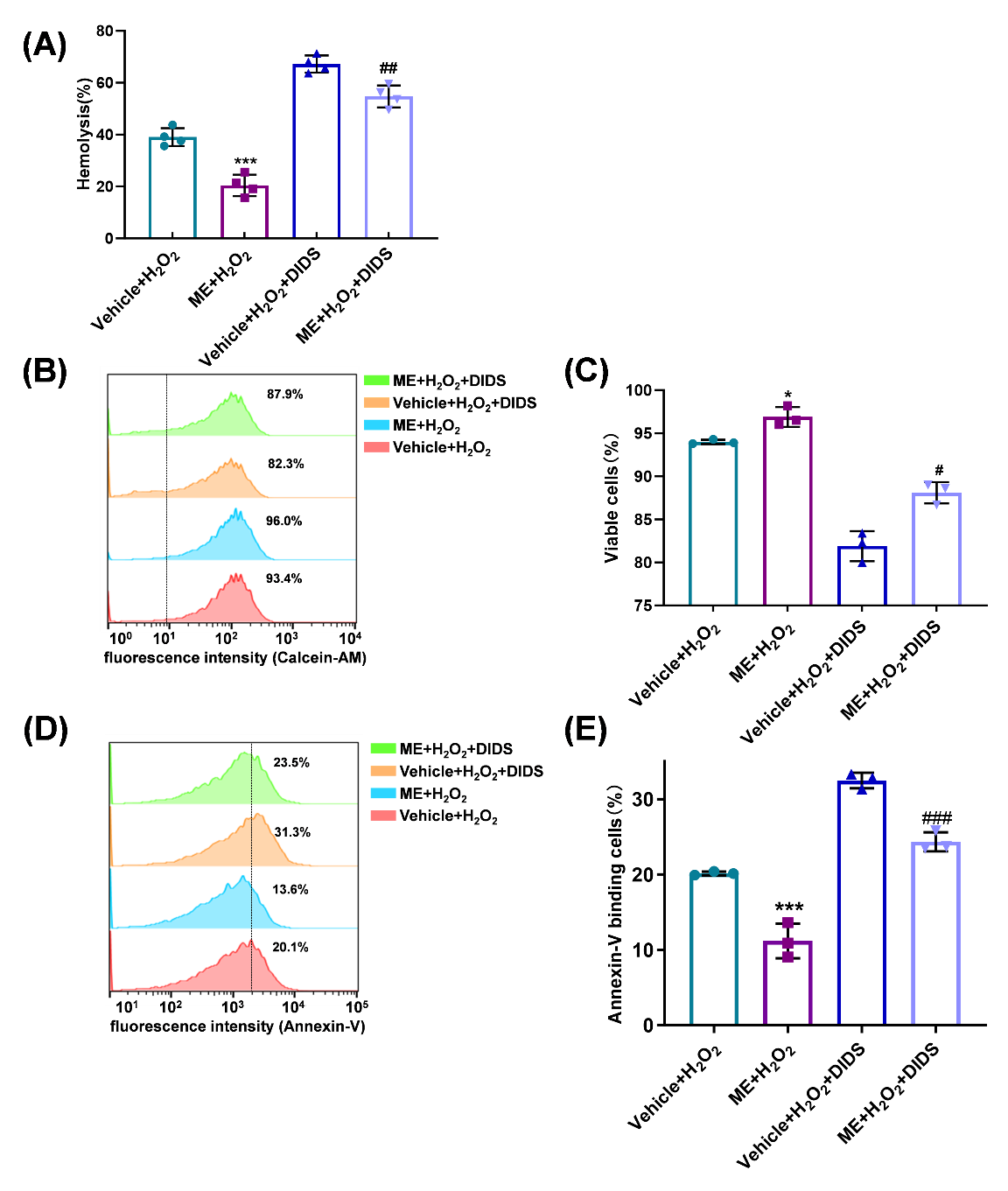
**

**Supplementary Figure 3. ME confers cytoprotection independently of Band 3 anion transport function.** Erythrocytes were preincubated with the Band 3 inhibitor 4,4′-diisothiocyanatostilbene-2,2′-disulfonate (DIDS; 10 µM) for 30 min to block anion exchange, followed by coincubation with Vehicle or 2 µM ME in the presence of 300 µM H_2_O_2_ for 24 h. **(A)** Hemolysis rate calculated as the percentage of total lysis. **(B)** Representative staggered histograms indicating cellular metabolic activity assessed by Calcein-AM staining. **(C)** Quantification of the percentage of metabolically active viable (Calcein-AM⁺) cells. **(D)** Representative staggered histograms indicating phosphatidylserine externalization assessed by Annexin V binding. **(E)** Quantification of the percentage of Annexin V⁺ (eryptotic) cells. Data are expressed as mean ± SD from independent biological replicates (n = 3–4). *P < 0.05, ***P < 0.001 *vs.* Vehicle + H₂O₂ group; ^#^P < 0.05, ^##^P < 0.01, ^###^P < 0.001 *vs.* Vehicle + H₂O₂ + DIDS group.

**Supplementary Figure 4.
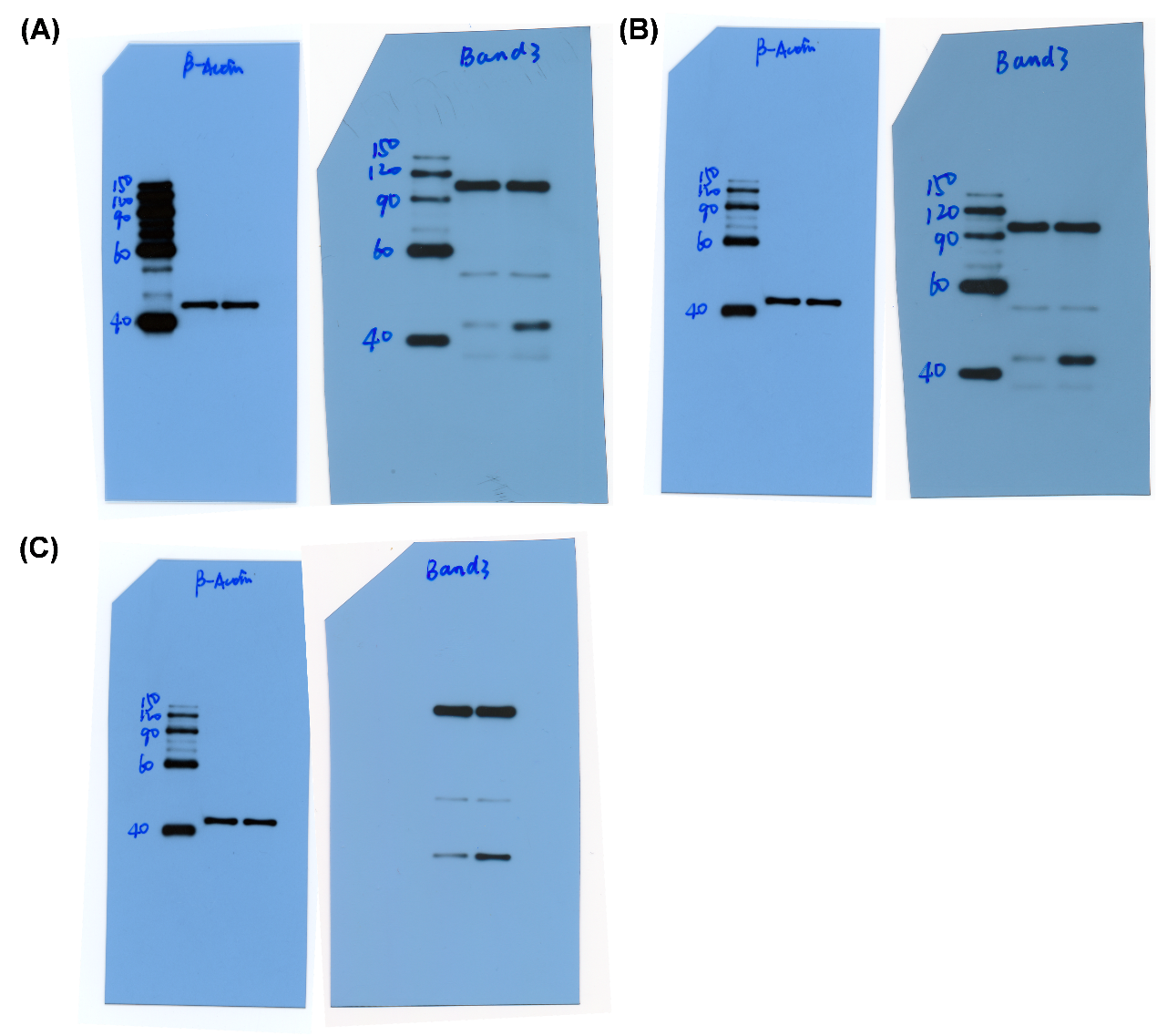
**

**Supplementary Figure 4. Original uncropped Western blot image corresponding to Figure 4B-D. (A).** Original uncropped image corresponding to Figure 4B shown in the main text. **(B).** Original uncropped image of the second biological replicate used for statistical analysis in Figure 4C-D. **(C).** Original uncropped image of the third biological replicate used for statistical analysis in Figure 4C-D.

**Supplementary Figure 5.
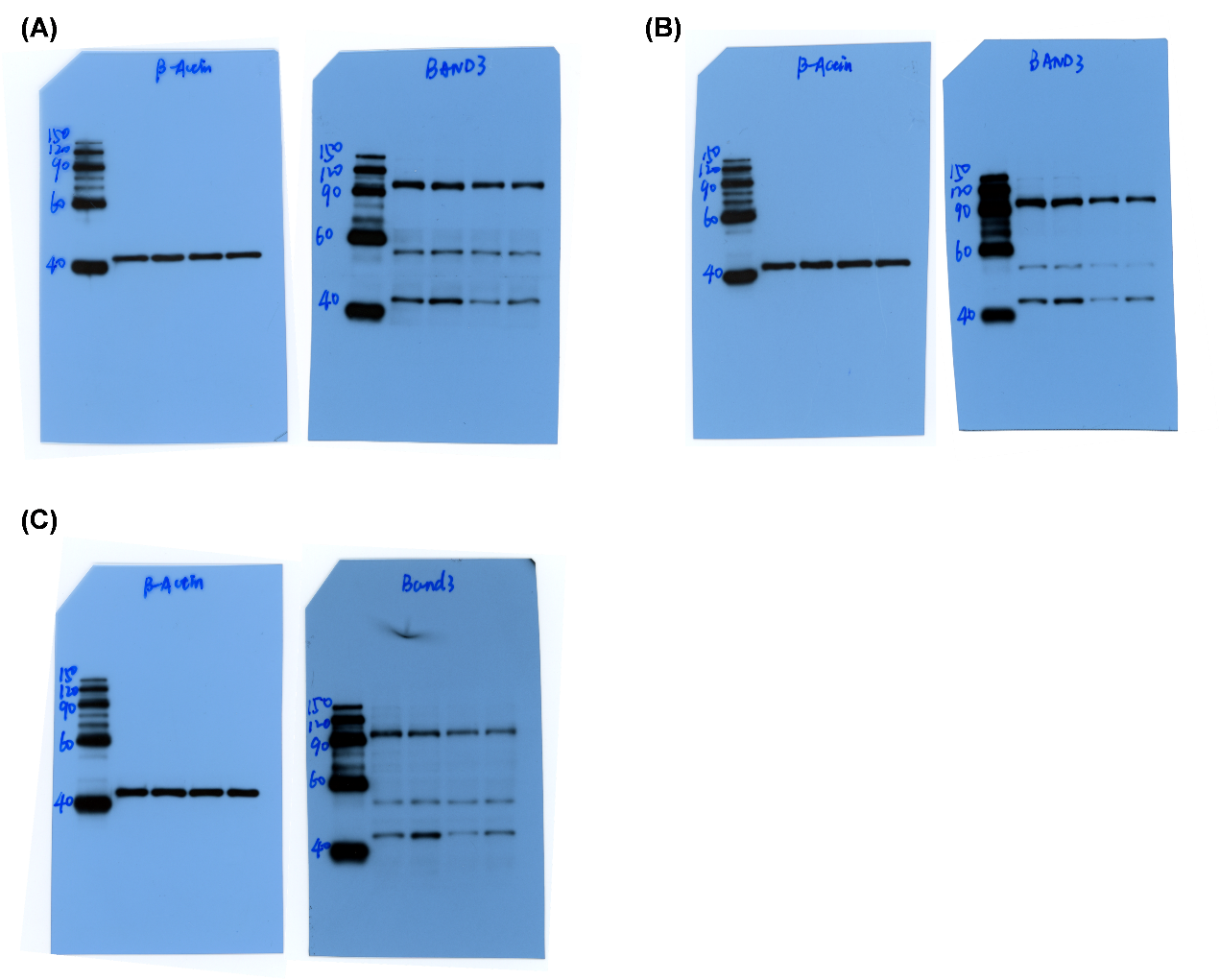
**

**Supplementary Figure 5.** **Original uncropped Western blot image corresponding to Figure 4F-H. (A).** Original uncropped image corresponding to Figure 4F shown in the main text. **(B).** Original uncropped image of the second biological replicate used for statistical analysis in Figure 4G-H. **(C).** Original uncropped image of the third biological replicate used for statistical analysis in Figure 4G-H.

**Supplementary Figure 6.
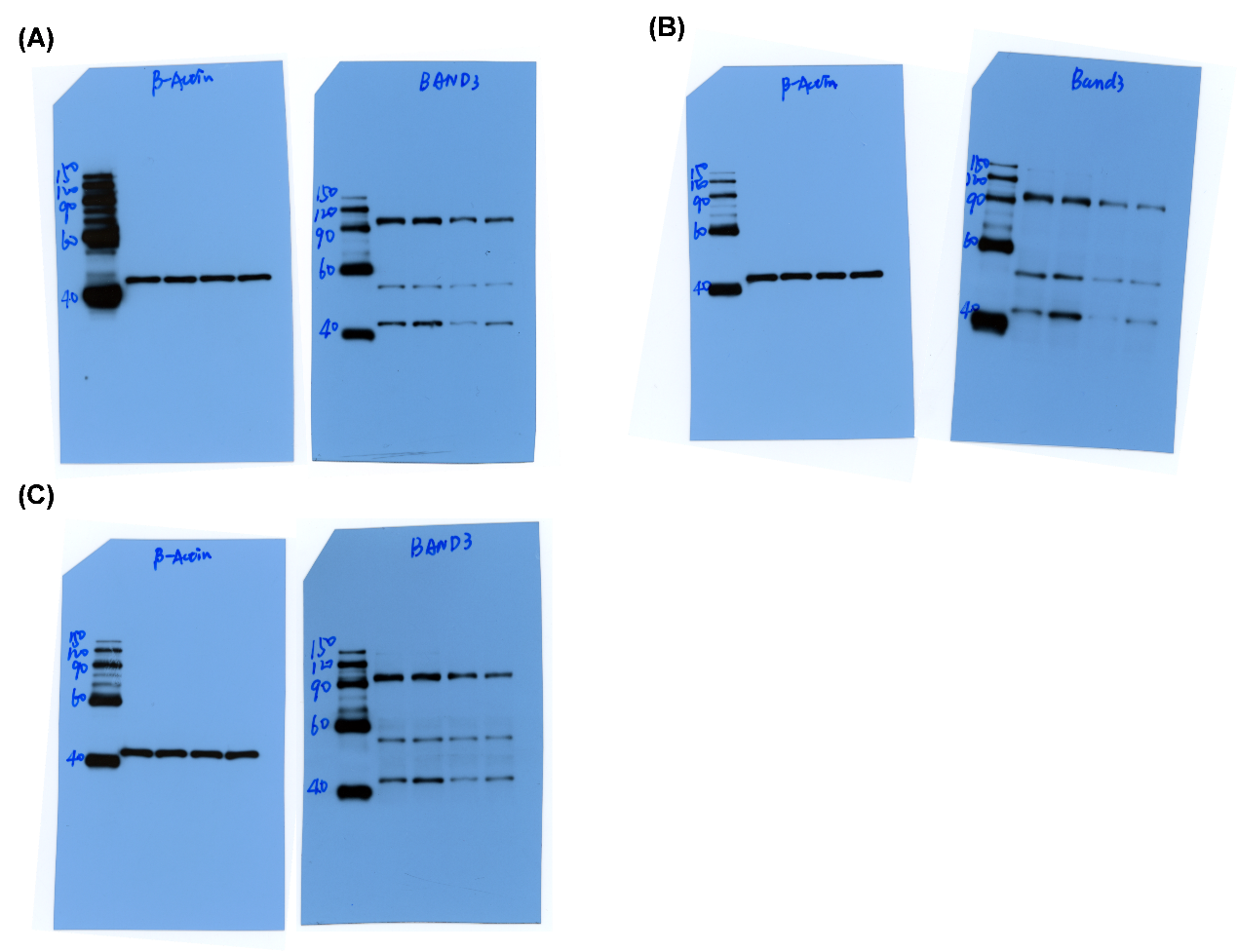
**

**Supplementary Figure 6. Original uncropped Western blot image corresponding to Figure 4I-K. (A).** Original uncropped image corresponding to Figure 4I shown in the main text. **(B).** Original uncropped image of the second biological replicate used for statistical analysis in Figure 4J-K. **(C).** Original uncropped image of the third biological replicate used for statistical analysis in Figure 4J-K.

**Table S1. Rate constants of SO4^2-^ uptake in human erythrocytes with or without 2μM ME.**

| **Group** | **Rate constants (min^-1^)** | **Time (min)** |
| --- | --- | --- |
| DMSO＋H_2_O_2_ | 0.029±0.004* | 35 |
| ME＋H_2_O_2_ | 0.04±0.003***, ^##^ | 25 |
| DIDS＋H_2_O_2_ | 0.020±0.006 | 50 |

*:p<0.05, ***: p<0.001, significant versus DIDS + H_2_O_2_; ##: p<0.01, significant versus DMSO+ H_2_O_2_; N＝4.

**Table S2. Rate constants of SO4^2-^ uptake in human erythrocytes with or without DIDS.**

| **Group** | **Rate constants (min^-1^)** | **Time (min)** |
| --- | --- | --- |
| DMSO＋H_2_O_2_ | 0.031±0.003 | 32 |
| ME＋H_2_O_2_ | 0.047±0.003^**^ | 21 |
| DMSO+ DIDS＋H_2_O_2_ | 0.017±0.004^**^ | 56 |
| ME+ DIDS＋H_2_O_2_ | 0.029±0.003^$$$, ##^ | 34 |

**p<0.01 significant versus DMSO＋H_2_O_2_; ## p<0.01 significant versus ME＋H_2_O_2_; $$$ p<0.001 significant versus DMSO＋DIDS＋H_2_O_2_; N＝4.

**Table S3. Rate constants of SO4^2-^ uptake in human erythrocytes with or without 3AT.**

| **Group** | **Rate constants (min^-1^)** | **Time (min)** |
| --- | --- | --- |
| DMSO＋H_2_O_2_ | 0.031±0.003 | 33 |
| ME＋H_2_O_2_ | 0.04±0.003**,### | 25 |
| DMSO+ 3AT＋H_2_O_2_ | 0.021±0.002*** | 48 |
| ME+ 3AT＋H_2_O_2_ | 0.024±0.002^$$^ | 41 |
| DIDS＋H_2_O_2_ | 0.017±0.003 | 59 |

**p<0.01 significant versus DMSO; ## p<0.01 significant versus ME＋3AT; $$ p<0.01 significant versus DMSO＋3AT; N＝4.
